# GFAP^+^ FOXF2^+^ ependymal cells promote blood–brain barrier repair via DLL4–NOTCH signaling after neural injury

**DOI:** 10.1073/pnas.2520352123

**Published:** 2026-03-24

**Authors:** Qi Xie, Hui Lu, Xiaoman Wang, Siya Wu, Qian He, Mengqi Yuan, Shuang Zhang, Linlin Hu, Changxiong Gong, Xiaofeng Cheng, Yiliang Fang, Zhaoyou Meng, Yilong Wang, Sen Lin, Qingwu Yang

**Affiliations:** ^a^Department of Neurology, Second Affiliated Hospital, Army Medical University (Third Military Medical University), Chongqing 400037, China; ^b^Chongqing Institute for Brain and Intelligence, Guangyang Bay Laboratory, Chongqing 400064, China; ^c^Department of Obstetrics and Gynecology, Southwest Hospital of the Third Military Medical University (The First Affiliated Hospital of the Army Medical University), Chongqing 400038, China; ^d^Department of Neurology, Beijing Tiantan Hospital, Capital Medical University, Beijing 100070, China; ^e^Chinese Institute for Brain Research, Beijing 102206, China

**Keywords:** stroke, FOXF2, ependymal cells, blood–brain barrier repair

## Abstract

Ependymal cells in the adult ventricular-subventricular zone are increasingly recognized for functions extending beyond cerebrospinal fluid dynamics; however, their identity and functional specialization remain incompletely understood. While ependymal cells (EP) have been implicated in interactions with the stem cell niche and the vasculature, their role in repair processes following neural injury remains elusive. In this study, we employed region-specific single-cell transcriptomics of the subventricular zone (SVZ) and ipsilesional peri-infarct territory in mice to identify a distinct subpopulation of GFAP^+^ FOXF2^+^ EP that selectively expand within the SVZ after neural injury. Notably, these cells were absent from other brain regions. Immunohistochemical validation revealed characteristic ependymal features, including typical pinwheel architecture and expression of Foxj1 and β-catenin. Furthermore, the absence of the proliferation marker Ki67 and the resistance of this subpopulation to Ara-C-mediated ablation indicate that these cells do not possess proliferative properties. Conditional deletion of *Foxf2* in GFAP^+^ cells led to impaired endothelial junction integrity and increased blood–brain barrier (BBB) permeability. In contrast, overexpression of *Foxf2* via GFAP promoter-driven adeno-associated virus delivery enhanced vascular repair and facilitated functional recovery. Mechanistically, these GFAP^+^ FOXF2^+^ EP secrete exosomal DLL4, which was associated with enhanced NOTCH pathway activity and restoration of BBB function. While the mechanism linking this limited cell population to the broad reparative effect, particularly the complete signaling amplification cascade, remains to be fully elucidated, these findings identify a subset of EP that contributes to BBB repair.

## Significance

Ependymal cells (EP) in the adult ventricular-subventricular zone (V-SVZ) are recognized for diverse functions beyond cerebrospinal fluid (CSF) dynamics, yet their identity, specialization, and roles in niche interactions and postinjury repair remain poorly understood. Our study identifies distinct injury-responsive ependymal subpopulations, highlighting GFAP^+^ FOXF2^+^ cells as a pivotal subset following stroke. Conditional deletion of *Foxf2* in GFAP^+^ EP results in markedly aggravated blood–brain barrier (BBB) disruption, demonstrating their indispensable role in BBB repair mediated through DLL4-dependent Notch signaling. These findings underscore GFAP^+^ FOXF2^+^ EP as a compelling therapeutic target for enhancing BBB integrity in the context of neural injury.

Ependymal cells (EP) are glial cells that form an epithelium lining the inner surfaces of the walls of the brain ventricles and the central canal of the spinal cord. They are located at the interface between cerebrospinal fluid (CSF) and the brain parenchyma and play a crucial role in the formation of the brain-CSF barrier (1). The apical ciliary clusters of EP beat in a coordinated manner to regulate the unidirectional flow of CSF. Moreover, EP can secrete cytokines and other signaling molecules, providing nutritional support and contributing to immune and metabolic regulation (2). Indeed, alterations in multiciliated brain EP have been studied in neurodegenerative disease, autoimmunity, traumatic brain injury (TBI), and stroke (3, 4). Thus, EP play crucial roles in both physiological and pathological processes within the central nervous system.

Recent studies have revealed significant heterogeneity among EP, which can be explored from two key perspectives: cell morphology and gene expression profiles. EP are categorized into three subtypes on the basis of cilia number and regional localization: multiciliated EP (E1), which line the brain ventricles and are essential for maintaining brain homeostasis; a rare ependymal subpopulation (E2) identified in the lateral ventricles, characterized by only two cilia and unique basal bodies, forming a distinct biciliated epithelium that extends from the ventral third ventricle into the fourth ventricle; and uniciliated EP (E3), which are found in the floor of the third ventricle where apical profiles display only primary cilia, defining an additional uniciliated epithelium. While E2 and E3 cells exhibit different distribution patterns, they have similar functions and are suggested to correspond to subtypes of tanycytes (5, 6). Additionally, while EP lining the central canal of the spinal cord do not generate other cell types under physiological conditions, this population harbors cells with in vitro neural stem cell (NSC) properties in the adult spinal cord. In mice, EP generate mostly scar-forming astrocytes and a few oligodendrocytes (OG) following spinal cord injury (7, 8). Furthermore, recent advancements in single-cell RNA sequencing have revealed significant heterogeneity in gene expression profiles among EP (9–11), with distinct subpopulations emerging under various physiological and pathological conditions. These findings indicate that while EP may share certain common molecular features, they can also present diverse characteristics that reflect their functional adaptability and roles within the central nervous system, particularly in response to changing internal and external environments.

Despite these advances, several key questions regarding EP persist, particularly concerning their identity, neurogenic potential, proliferative capability, and functional roles. First, distinguishing EP from neighboring adult B cells and neural stem cells (NSCs) poses considerable challenges, especially as EP can express Glial Fibrillary Acidic Protein (GFAP) following injury, complicating efforts to differentiate them from NSCs, which share a common developmental origin (12). Additionally, their neurogenic potential and proliferative capability are still highly debated. In the early stages of single-cell sequencing, a subset of EP was discovered, suggesting the presence of a dormant NSC state that can be activated under conditions such as brain injury, where local levels of angiogenic factors significantly increase (13). However, subsequent studies have indicated that EP do not behave as NSCs or progenitors in vitro or in vivo. While specific subsets of EP may exhibit unique characteristics, they do not represent EP as a whole (14). Furthermore, EP are recognized as important regulators of the neural environment; they release extracellular signals that modulate the activity of adjacent stem cells and influence pericyte and endothelial cell functions (15). However, the specific mechanisms through which EP exert these effects require further investigation.

This study employed single-cell RNA sequencing of the subventricular zone (SVZ) following stroke, identifying GFAP^+^ FOXF2^+^ EP as a critical population regulating blood–brain barrier (BBB) integrity. These cells promote BBB repair through the secretion of Delta-like ligand 4 (DLL4). This research significantly advances our understanding of ependymal cell biology and highlights potential therapeutic targets for BBB restoration in stroke recovery.

## Results

### Single-Cell Transcriptome Sequencing of the SVZ Region after Stroke.

**Fig. 1.**
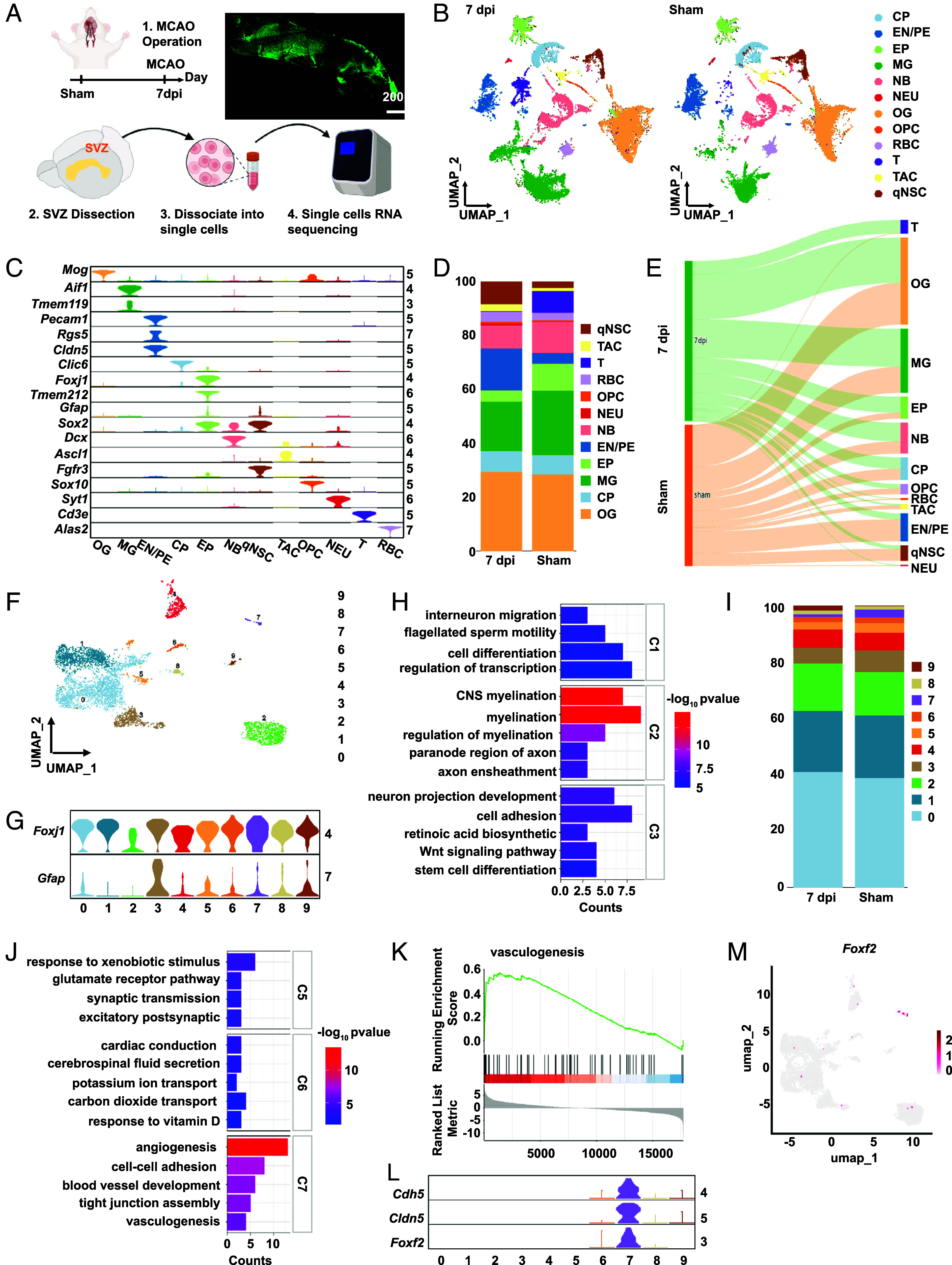
Single-cell transcriptomic profiling of the SVZ region following stroke. (*A*) Experimental workflow for single-cell RNA analysis in the SVZ. The SVZ samples used for single-cell sequencing were obtained from three different mice in each group. (*B*) UMAP plot illustrating the distribution of individual cell subsets. (*C*) Violin plot displaying cell type–specific marker expression in the clusters. (*D*) The proportional abundance of each cell subgroup. (*E*) Sankey diagram illustrating the differences in both cell numbers and cell proportions between the sham and 7 dpi groups for each cell type. (*F*) The ependymal cell subset was further subdivided to characterize the cell subpopulations. (*G*) Violin plot of *Foxj1* and *Gfap* expression, with *Gfap* categorized into low expression (clusters 1 and 2), high expression (cluster 3), and medium expression in other clusters. (*H*) Enrichment analysis of the biological processes associated with the high- and low-expression *Gfap* subpopulations. (*I*) Comparison of the proportions of ependymal cell subpopulations between the sham group and the 7 dpi group. (*J*) BP analysis of three ependymal cell subpopulations (clusters 5, 6, and 7) whose proportions increased at 7 dpi. (*K*) GSEA enrichment analysis identifying core genes that regulate angiogenesis pathways in cluster 7 (C7). (*L*) Violin plot validating the expression specificity of key angiogenesis molecules in ependymal cell subpopulations. (*M*) The scatter plot confirms that *Foxf2* is highly expressed only in the ependymal cell subpopulation C7.

A mouse model of ischemic stroke with middle cerebral artery occlusion (MCAO) was employed, as it provides a broad range of injury etiologies and is an excellent system for studying NSC recruitment for repair (14). Specifically, the MCAO model involves unilateral occlusion for 90 min followed by reperfusion. Following established protocols (16), we microdissected the SVZ with intact cytoarchitecture (*SI Appendix*, Fig. S1*A*) and performed single-cell transcriptomic profiling to characterize the cellular diversity within this specialized niche (Fig. 1*A*). Subsequent analysis of 60,565 quality-controlled cells in Seurat included projections visualized in Uniform Manifold Approximation and Projection (UMAP) space. The sham group included 28,380 cells, and the 7-day postinjury (7 dpi) group included 32,185 cells, with samples pooled from three mice in each group for analysis (Fig. 1*B*). Cluster annotation via FindAllMarkers (17) identified 12 distinct cell types, including oligodendrocytes (OG, *Mog*), oligodendrocyte progenitor cells (OPC, *Sox10*), microglia (MG, *Tmem119* and *Aif1*), endothelial cells and pericytes (EC/PC, *Cldn5, Pecam1,* and *Rgs5*), the choroid plexus (CP, *Clic6*), (EP, *Tmem212* and *Foxj1*), neuroblasts (NB, *Dcx*), T cells (*CD3e*), red blood cells (RBC, *Hba-a1*), and mature neurons (NEU, *Syt1*). *Sox2* is utilized as a marker for NSCs. *Ascl1* (also known as Mash1) is subsequently utilized to identify transit-amplifying cells (type C cells, TAC), whereas fibroblast growth factor receptor 3 is used to identify quiescent neural stem cells (qNSCs) (18). Notably, *Gfap*, which is typically regarded as a marker for astrocytes, was also identified in EP (Fig. 1*C* and *SI Appendix*, Table S1).

To confirm the changes in cell types in the SVZ region after stroke, we compared the cell proportions between the sham and 7-dpi groups (Fig. 1*D*). The results revealed minimal differences in the proportion of OG, whereas the proportions of MG, NB, and T cells significantly increased in the 7 dpi group. In contrast, the number of qNSCs, as well as endothelial and pericyte cells, significantly decreased, which aligns with previously reported changes in cell types following stroke (19–21). This finding is further supported by the changes observed in the Sankey diagram (Fig. 1*E*). Notably, the population of EP also markedly increased in the 7 dpi group. The heterogeneity of EP has been less explored in the context of neural injury, warranting further investigation.

First, MG were subdivided into distinct 13 subclusters (*SI Appendix*, Fig. S1 *B* and *C*), showing marked activation of immune-related pathways (*SI Appendix*, Fig. S1*D*). Although overall subcluster numbers increased, cluster 0 decreased (*SI Appendix*, Fig. S1*C*), suggesting a homeostatic role. Trajectory analysis showed cluster 0 differentiating into clusters 1, 3, and 4 (*SI Appendix*, Fig. S1*E*), with cluster 1 markedly expanded at 7 dpi, indicating its role in activation. Heatmap analysis revealed high expression of homeostatic markers *Tmem119* and *Siglech* in cluster 0, whereas activation markers *Spp1* and *Ms4a7* were enriched in clusters 1 and 2 (*SI Appendix*, Fig. S1 *F–H*), consistent with previous reports (20, 22).

We reclustered NB (*SI Appendix*, Fig. S2 *A* and *B*), and a Sankey diagram revealed minimal changes in cluster 1, a decrease in cluster 5, and expansion of the remaining subpopulations (*SI Appendix*, Fig. S2*C*). Markers from cluster 0 were widely expressed in other subpopulations, indicating general neuroblast markers. Cluster 1 expressed proliferation markers *Top2a* and *Ki67* (*SI Appendix*, Fig. S2*D*), suggesting high proliferative potential. Clusters 2 and 3 expressed *Mog* and microglial activation markers (*C1qc, Ctss*). Clusters 4 and 5 were enriched in metabolism-related markers (*SI Appendix*, Fig. S2*E*). Trajectory analysis showed cluster 1 as the origin of transdifferentiation, differentiating into clusters 0 and 5 (*SI Appendix*, Fig. S2*F*). Pseudotime analysis and heatmaps identified key molecules driving cluster 1’s differentiation (*SI Appendix*, Fig. S2 *G* and *H*), revealing future research directions.

We subsequently subdivided the ependymal cell population into ten subclusters (clusters 0 to 9) (Fig. 1*F* and *SI Appendix*, Table S2), all of which presented high *Foxj1* expression, confirming their identity as EP (23). Notably, *Gfap* expression varied among these subclusters: clusters 1 and 2 presented low expression, cluster 3 presented high expression, and the other clusters presented moderate expression (Fig. 1*G*). We then analyzed the functional differences among clusters 1, 2, and 3 to assess how variations in *Gfap* expression correlate with cellular functions. The results revealed that clusters 1 and 2 were associated primarily with myelination and cellular transcription, whereas cluster 3 was linked mainly to cell differentiation and adhesion (Fig. 1*H*). The relationship between these signaling pathway differences and *Gfap* expression requires further investigation.

Furthermore, we analyzed the proportional changes in ependymal subclusters and found that the differences between the two groups were minimal for clusters 0 to 4; however, clusters 5, 6, and 7 presented increases in the 7 dpi group (Fig. 1*I*). Therefore, we conducted a functional clustering comparison of these three subclusters and found that C7 was highly associated with angiogenesis-related pathways (Fig. 1*J*), which differed from previously reported functions of EP. Consequently, we further analyzed the characteristics of C7. We performed Gene Set Enrichment Analysis (GSEA) (24) to identify key molecules regulating angiogenesis pathways in C7 (Fig. 1*K*). The top three ranked molecules were *Cdh5*, *Cldn5*, and *Foxf2*, which were highly expressed specifically in C7 (Fig. 1*L*). *Cdh5* and *Cldn5* are commonly recognized as markers of endothelial and pericyte identity. *Foxf2* is essential for brain pericyte differentiation and development, as well as for the maintenance of the BBB. It is expressed in brain PC, and *Foxf2*^−/−^ embryos present severe conditions, such as intracranial hemorrhage, perivascular edema, and a compromised BBB. In adult mice, inactivation of *Foxf2* leads to BBB breakdown (25). Additionally, *Foxf2* is also expressed in the brain vascular endothelium and plays a role in BBB maturation (26). Deletion of *Foxf2* in adult mice led to the occurrence of microhemorrhages (27). We examined the scatter plot of *Foxf2* expression and confirmed that it was exclusively expressed in C7 (Fig. 1*M*). However, the expression of *Foxf2* in GFAP-positive EP and its associated functions have yet to be documented.

To resolve potential identity ambiguities arising from shared markers between ependymal and stem cell populations, we conducted systematic expression profile comparisons that placed C7 unequivocally within the ependymal lineage, which was supported by external datasets. Our analysis confirmed that C7 exhibits significant similarity to canonical ependymal signatures while demonstrating marked dissimilarity from neural precursor profiles (NB and NSCs) across published single-cell references (28–31) (*SI Appendix*, Fig. S3 *A*–*H*). Furthermore, cross-dataset validation revealed that conserved ependymal subpopulations not only presented transcriptional congruence with C7 but also demonstrated parallel proangiogenic functionality, consistently maintaining FOXF2 expression across an independent single-cell atlas (32) (*SI Appendix*, Fig. S3 *I*–*K*). Importantly, our comparative analysis with the latest sequencing data from TBI models revealed GFAP^+^ FOXF2^+^ double-positive cell expression signatures within the ependymal subcluster (10) (*SI Appendix*, Fig. S3 *L* and *M*). These findings establish GFAP^+^ FOXF2^+^ EP as a transcriptomically conserved population that is detectable across multiple single-cell atlases. However, their comprehensive functional significance warrants further investigation.

### Validation of Expression Profiles Characterizing Subpopulations of Cells with Angiogenic Functions in the Ventricular-Subventricular Zone (V-SVZ).

**Fig. 2.**
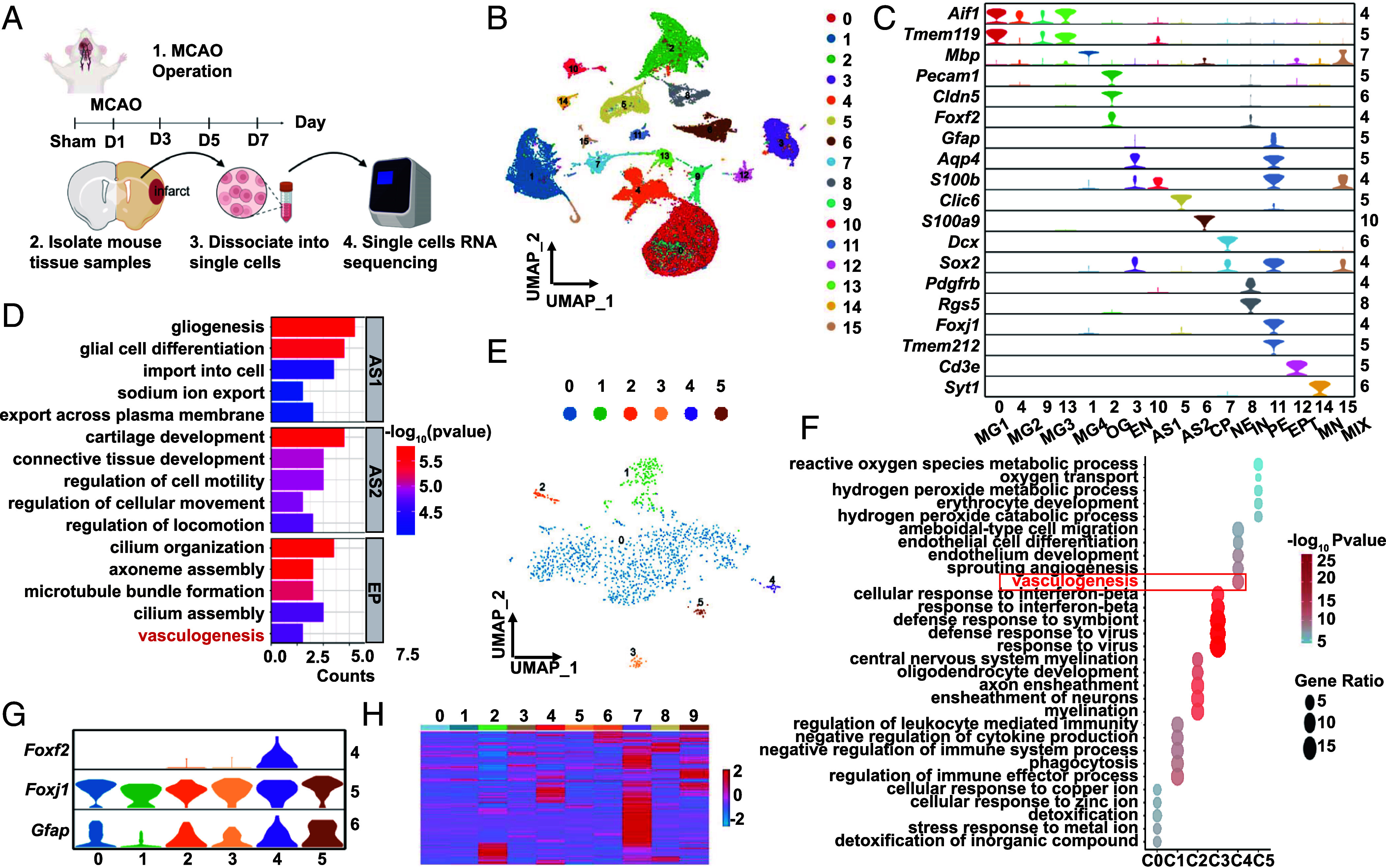
Single-cell transcriptome sequencing of the infarcted region after stroke. (*A*) Experimental workflow for single-cell RNA analysis of infarcted tissue after stroke. The infarcted samples used for single-cell sequencing were obtained from three different mice in each group. (*B*) UMAP plot showing the distribution of individual cell subsets. (*C*) Violin plot displaying cell type–specific marker expression in the clusters. (*D*) BP analyses for astrocyte and ependymal clusters. (*E*) The ependymal subset was further subdivided to characterize the cell subsets associated with angiogenesis. (*F*) Enrichment analysis of the biological processes of each cell subset after subdivision indicates that cluster 4 is predominantly involved in the angiogenesis pathway. (*G*) The violin plot further shows that C4 presented significantly elevated expression levels of *Foxj1*, *Foxf2*, and *Gfap*. (*H*) The expression profile of ependymal cell subpopulation cluster 4 in Fig. 2 is most similar to that of ependymal cell subpopulation C7 in Fig. 1.

While our V-SVZ scRNA-seq data revealed sparse angiogenic GFAP^+^ FOXF2^+^ EP in the stroke model (Fig. 1), the region-specific sampling approach left unresolved whether these cells remain restricted to the V-SVZ or migrate to infarcted territories. To resolve this spatial uncertainty, we performed whole-hemisphere scRNA-seq of the infarcted brain (Fig. 2*A*). This comprehensive approach simultaneously accomplishes two objectives: confirming the presence of these cells within the V-SVZ and excluding their existence in non-SVZ regions. Thus, we extracted cells from the infarcted side at 1, 3, 5, and 7 d postischemia and conducted single-cell transcriptome sequencing to elucidate the cellular diversity in that area (Fig. 2*A*). Following quality control, 81,609 cells were integrated and clustered via Seurat, with the resulting clusters annotated according to the established identities defined in Fig. 1*C*. This yielded 16 distinct cellular populations, including OG, MG, and EC, as visually demarcated (Fig. 2 *B* and *C* and *SI Appendix*, Table S3). Furthermore, our violin plot analysis of *Gfap* and *Foxf2* expression revealed distinct spatial distributions: *Gfap* was highly expressed in ependymal cell cluster 11 (C11), whereas *Foxf2* was concentrated primarily in EC (cluster 2) and PC (cluster 8). These findings indicate the absence of significant *Gfap* and *Foxf2* coexpression on the infarct side (Fig. 2 *B* and *C*). The negligible *Foxf2* expression in ependymal clusters at the current resolution suggests that this gene may mark discrete subpopulations, which requires further ependymal subclustering to resolve expression patterns.

Clustering analysis showed that MG and astrocytes had significant heterogeneity. They were distributed across multiple subpopulations (Fig. 2*D* and *SI Appendix*, Fig. S4*A*). In our recently published article (33), we analyzed the heterogeneity of astrocytes and MG after stroke. We also clarified how their interactions regulate stroke pathology. Therefore, we do not elaborate on the heterogeneity of these two cell types here.

Building upon our prior sequencing data revealing distinct ependymal subpopulations, we conducted selective repartitioning of C11 EP to evaluate the recurrence of analogous functional subtypes and characteristic molecular signatures. This subcluster-level validation directly tests the reproducibility of our initial findings. Notably, C11 expressed markers of both astrocytes (*S100β*, *Aqp4*, and *Gfap*) and EP (*Tmem212*) (Fig. 2*C*), suggesting potential localization in the V-SVZ, where EP are found. The biological process (BP) analysis of C11 revealed a significant enrichment of vasculogenesis-related signals, indicating a distinct pathway from those previously identified in astrocytes and EP (Fig. 2*D*).

Next, C11 was further subdivided to identify the molecular signatures of subgroups associated with vasculogenesis. The findings indicated that C11 could be categorized into six distinct subgroups (Fig. 2*E* and *SI Appendix*, Fig. S4*B* and Table S4), all of which expressed the ependymal cell marker *Foxj1* (*SI Appendix*, Fig. S4*C*), whereas each cluster presented distinct expression profiles (*SI Appendix*, Fig. S3*B*). Among these newly identified subpopulations, cluster 4 demonstrated significant enrichment of angiogenesis-related functions (Fig. 2*F*), indicating the presence of an angiogenesis-related cell population and angiogenesis-related genes in the V-SVZ (*SI Appendix*, Fig. S4*C*). Additionally, the violin plot demonstrated markedly high coexpression of *Foxj1*, *Foxf2,* and *Gfap* in cluster 4 (Fig. 2*G* and *SI Appendix*, Fig. S4*C*), revealing a molecular signature strikingly similar to the functionally specialized ependymal subcluster previously identified in Fig. 1*K*. In addition, when the gene expression profile of cluster 4 was compared with that of the ten subpopulations of the ependyma identified in Fig. 1*F*, the results revealed that it was most similar to the expression profile of C7 (Fig. 2*H*), which also confirms the reliability of our data.

In comparison with the previously characterized E1, E2, and E3 markers (5, 6), our analysis found that the target subpopulation predominantly expresses the E1/E2 marker CD24, while the E2-specific marker FEZF2 is absent. This suggests that the identified cells are primarily E1-type EP (*SI Appendix*, Fig. S4*D*). Additionally, the distribution of E1 cells in the lateral ventricle aligns with our sampling region (5), further supporting this classification.

### Validation of the GFAP^+^ FOXF2^+^ Ependymal Cell Subpopulation after Injury.

Using the MCAO model, we validated the ependymal characteristics of GFAP^+^ FOXF2^+^ cells. FOXF2 showed vascular-like staining colocalized with the endothelial marker CD31 in the cortex and striatum (*SI Appendix*, Fig. S5*A*). GFAP expression was markedly elevated in the peri-infarct region and SVZ (*SI Appendix*, Fig. S5*B*). Critically, GFAP^+^ FOXF2^+^ double-positive cells were found to be exclusively localized to the V-SVZ region, with no expression detected in other SVZ areas, cortex, hippocampus, or peri-infarct regions (*SI Appendix*, Fig. S5 *B* and *C*), confirming their site-specific expression.

**Fig. 3.**
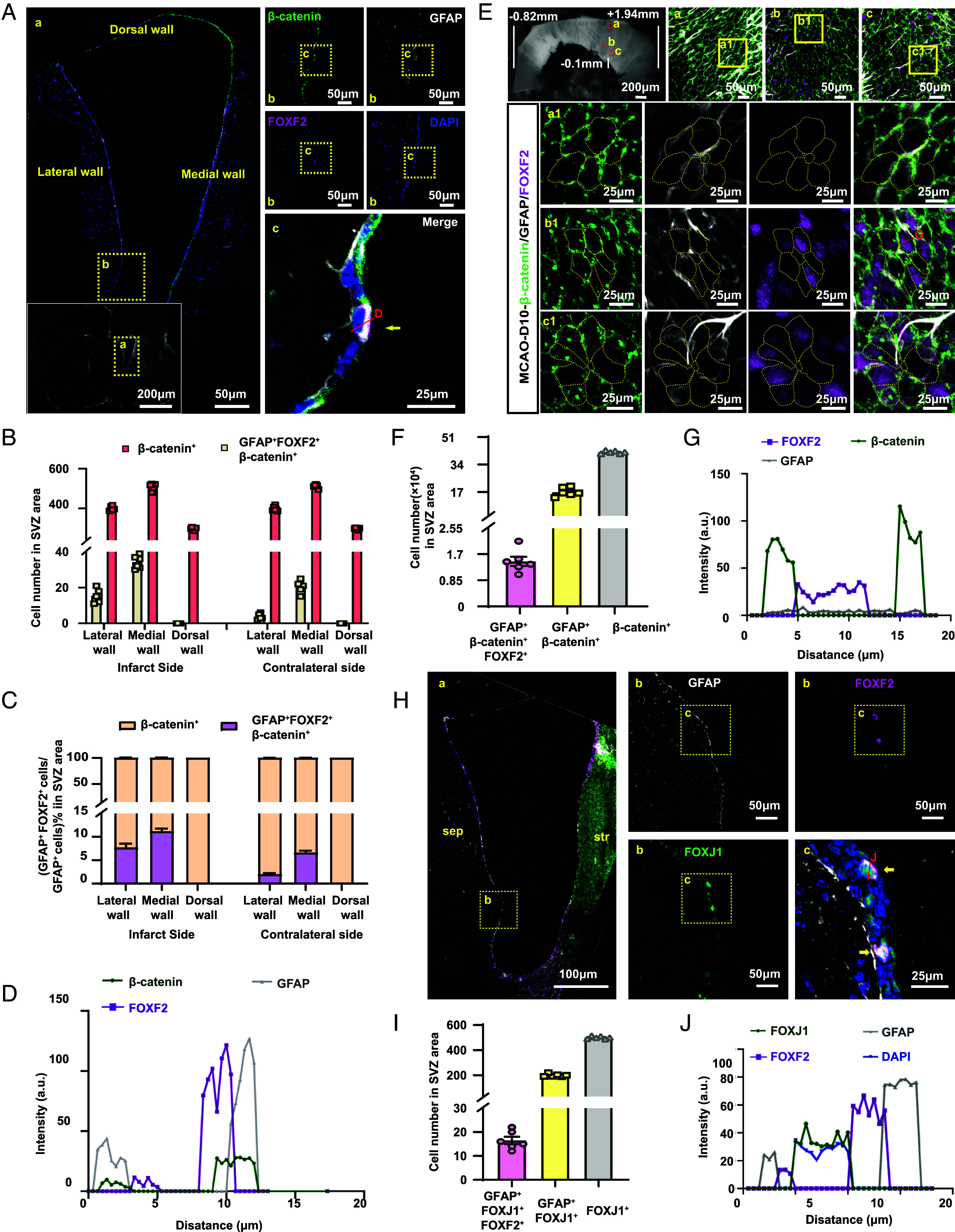
GFAP^+^ FOXF2^+^ cells were identified as a distinct subpopulation of EP. (*A*–*D*) Quantification of GFAP^+^ FOXF2^+^ β-catenin^+^ EP and β-catenin^+^ cells, including their proportions within the total ependymal cell population and their spatial distribution. (*E*) FOXF2 was distributed in the cytoplasm, GFAP was expressed at low levels throughout the entire section, and β-catenin exhibited a pinwheel architecture. (*F*) The number of cells expressing GFAP^+^ FOXF2^+^ β-catenin^+^, GFAP^+^ β-catenin^+^ and β-catenin^+^ cells in the entire SVZ area was determined. (*G*) The spatial location of protein expression in cells. (*H*) FOXF2 is distributed in the cytoplasm of these cells, whereas FOXJ1 is expressed in the nucleus. (*I*) The numbers of cells expressing GFAP^+^FOXF2^+^FOXJ1^+^, GFAP^+^FOXJ1^+^, and FOXJ1^+^ in the SVZ region were determined; n = 6 mice per group. (*J*) The spatial location of protein expression in cells.

We further investigated whether GFAP^+^ FOXF2^+^ cells colocalized with β-catenin-positive cells, a marker of EP. On the basis of established histological criteria, we partitioned the V-SVZ into three distinct regions: the dorsal wall, medial wall, and lateral wall. At 7 d poststroke, characterization of GFAP^+^ FOXF2^+^ EP revealed that their expression was confined to the medial and lateral walls of the ventricular system in the infarcted hemisphere, with no detection in the dorsal wall (Fig. 3*A*). These double-positive cells represented 5 to 10% of the ependymal population (β-catenin-positive cells) within the medial and lateral walls. A mirrored expression pattern was observed in the contralateral hemisphere, although the expression levels were significantly attenuated compared with those on the infarct side, constituting only 1 to 4% of the EP in the corresponding regions (Fig. 3 *B*–*D*).

Considering the distinctive pinwheel architecture of EP, we performed whole-mount SVZ immunostaining to validate GFAP^+^ FOXF2^+^ expression. The MCAO models showed marked expression of GFAP^+^ FOXF2^+^ cells within the pinwheel domains. Quantification revealed that GFAP^+^ FOXF2^+^ cells constituted approximately 5% of the total ependymal population (Fig. 3 *E–G*).

Furthermore, we validated the GFAP^+^ FOXF2^+^ expression profile via the expression of FOXJ1, a canonical ependymal marker. FOXJ1 expression was restricted to ventricular niches, whereas GFAP^+^ FOXF2^+^ cells displayed spatial patterns consistent with our previous findings. These results confirmed that GFAP^+^ FOXF2^+^ cells represent a distinct ependymal subpopulation induced postinjury, constituting 5 to 10% of the total ependymal cell pool (Fig. 3 *H* and *I*).

### Spatiotemporal Expression Characteristics of GFAP^+^ FOXF2^+^ EP.

**Fig. 4.**
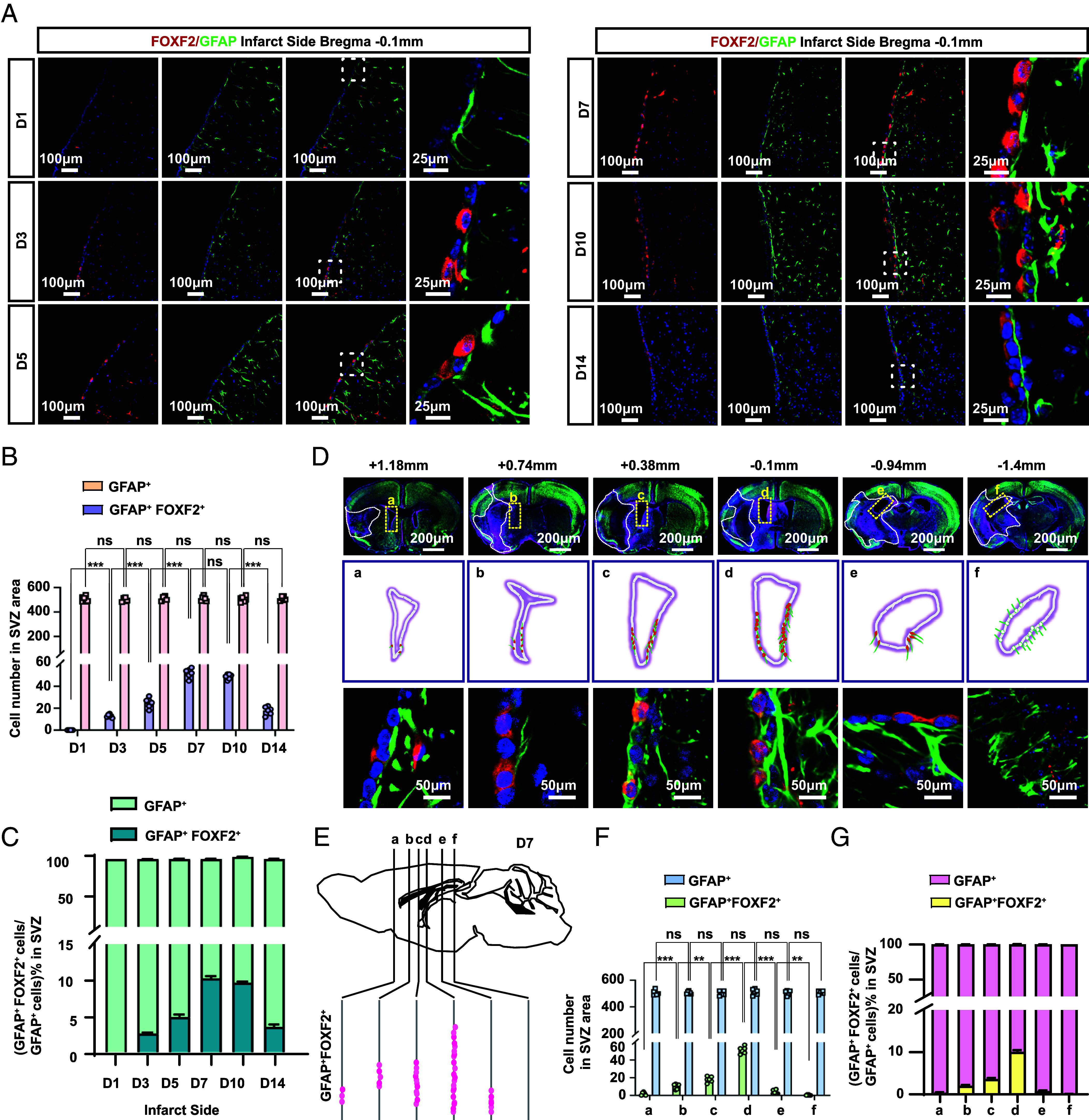
Spatiotemporal expression characteristics of the GFAP^+^ FOXF2^+^ ependymal subpopulation after stroke. (*A*) Representative immunofluorescence images of FOXF2 and GFAP expression on the infarct side of the V-SVZ region at different time points after I/R. n = 6, magnification 400×; the dotted boxes indicate the colabeled areas of the cells that are magnified. (*B* and *C*) Quantitative analysis of the cell counts and proportions of GFAP^+^ FOXF2^+^ cells in the SVZ region at different time points after I/R. (*D*) A serial section of the entire lateral ventricular region was stained to observe the spatial characteristics of FOXF2 expression in GFAP^+^ cells. The schematic diagram displays the distribution of GFAP^+^ FOXF2^+^ positive cells in this field of view. (*E*) Distribution map of GFAP^+^ FOXF2^+^ cells in the V-SVZ. The black lines represent coronal sections of three mice from rostral to caudal levels. Each pink dot corresponds to the location of a single positive cell. (*F*) Quantification of the positive cells in (*E*). (*G*) Quantitative comparison of the proportion of Foxf2-expressing cells to all GFAP-positive cells in the SVZ region. Data are shown as mean ± SEM. Significance was calculated using paired two-tailed Student’s *t* test. *P < 0.05, **P < 0.01, ***P < 0.001; ns, not significant.

We investigated the spatiotemporal expression profile of GFAP^+^ FOXF2^+^ EP via the MCAO model. The results revealed that in the V-SVZ region, the number of GFAP^+^ cells did not significantly change after I/R, whereas the number of GFAP^+^ FOXF2^+^ cells on the infarcted side began to increase 3 d after I/R, peaked 7 d, and then gradually declined (Fig. 4 *A* and *B*). This finding is consistent with the previously reported timelines of angiogenesis following MCAO (34). Moreover, FOXF2 expression represented only approximately 10 to 20% of the GFAP^+^ cell population in the V-SVZ, suggesting that it constituted a specific subset of GFAP^+^ cells (Fig. 4*C*). To quantify the changes in the number of GFAP^+^ FOXF2^+^ cells in the V-SVZ, we counted the number of double-positive cells in each layer after serial sectioning of the entire V-SVZ (Fig. 4*D*) via the method reported in the literature (35). The double-positive cells were most prevalent adjacent to the bregma, +0.38 to −0.1 mm from the V-SVZ (Fig. 4 *E–G*). These findings confirm the comprehensive spatiotemporal distribution profile of GFAP^+^ FOXF2^+^ EP postinjury.

Upon confirming increased GFAP^+^ FOXF2^+^ ependymal cell expression postinjury, we investigated their association with cell proliferation via Ki67 immunolabeling. The results revealed no colocalization between Ki67^+^ proliferating cells and the GFAP^+^ FOXF2^+^ population, indicating their nonproliferative status (*SI Appendix*, Fig. S6 *A* and *B*). Furthermore, cytosine arabinoside treatment to suppress cell proliferation in the SVZ region significantly reduced the number of Ki67-positive cells, whereas the population of GFAP^+^ FOXF2^+^ EP remained unaffected. These findings demonstrated that GFAP^+^ FOXF2^+^ cells were nonproliferative (*SI Appendix*, Fig. S6 *C* and *D*).

Moreover, we tested the number of GFAP^+^ FOXF2^+^ double-positive cells in other disease models. First, we used another model of cerebral ischemia, the photothrombotic model, because it can generate localized ischemic infarcts in the desired region (36). We created photothrombotic models with Bregma lengths of 1.18 mm (*SI Appendix*, Fig. S7 *A* and *B*) and 0 mm (*SI Appendix*, Fig. S7 *C* and *D*) to evaluate the effects of different injury locations on the number of GFAP^+^ FOXF2^+^ double-positive cells. The results revealed that the expression of these double-positive cells was mainly concentrated in the 0 mm Bregma group, and these cells were expressed primarily in the middle of the ventricle, with minimal expression at the edges. In contrast, almost no double-positive cells were detected in the photothrombotic model group at Bregma 1.18 mm (*SI Appendix*, Fig. S7 *E–G*). These results suggest that with a similar degree of injury severity, the closer the injury is to the V-SVZ region, the greater the number of double-positive cells. Similarly, the double-positive cells were also expressed in the V-SVZ and concentrated at Bregma −0.14 mm in the Alzheimer’s disease model (*SI Appendix*, Fig. S7 *H* and *I*). These findings collectively suggest that GFAP^+^ FOXF2^+^ ependymal cell expression is strongly correlated with the location and severity of injury.

### Deletion of the Foxf2 Gene in GFAP-Positive Cells Exacerbates BBB Damage after Stroke.

In addition to confirming the expression characteristics of GFAP^+^ FOXF2^+^ EP under pathological conditions, we also examined their expression under physiological conditions. Notably, these cells exhibited almost no expression in young mice (2 mo and 6 mo), whereas there was significant expression in middle-aged mice (12 mo), suggesting a potential relationship between these cells and aging (*SI Appendix*, Fig. S8 *A* and *B*). We constructed two types of knockout mice, *Foxj1*^Cre^; *Foxf2*^fl/fl^ and *Gfap^c^*^re^; *Foxf2*^fl/fl,^ to compare their phenotypes for any differences (*SI Appendix*, Fig. S8*C*). A comparative analysis of aged mice (12 mo old) revealed that the vascular leakage of both strains was significantly greater than that of the *Foxf2*^fl/fl^ controls, whereas no statistically significant difference was detected between the two strains. Importantly, in young mice (2 mo old), no groups presented evidence of vascular leakage, indicating that the observed vascular dysfunction developed with age (*SI Appendix*, Fig. S8 *D–G*). These results suggest that there is no marked difference in the vascular leakage phenotype between the two types of knockout mice.

**Fig. 5.**
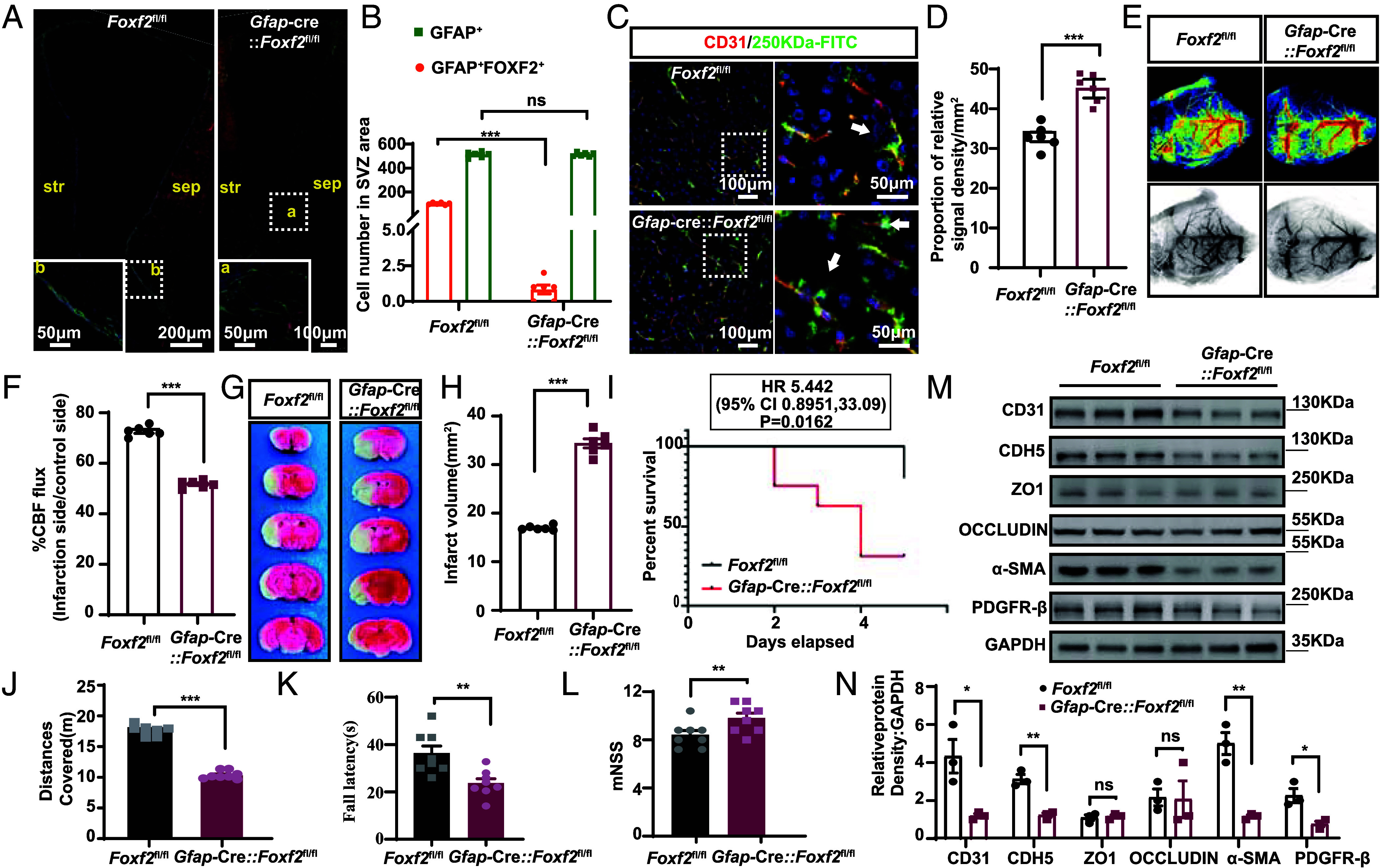
Deletion of *Foxf2* expression in GFAP-positive cells exacerbates BBB injury in mice after I/R. (*A* and *B*) Detection of GFAP^+^ FOXF2^+^ cell numbers after I/R in the *Foxf2*-CKO group to validate the knockdown efficiency, n = 6. (*C* and *D*) Quantitative comparison of 250 kDa Fluorescein Isothiocyanate (FITC) leakage (green) and CD31 staining in *Foxf2*-CKO mice following I/R., n = 6. (*E* and *F*) Representative images and quantification of laser Doppler measurements of blood flow signals in control (*Foxf2*^fl/fl^) and *Foxf2*-CKO mice following I/R, n = 6. (*G* and *H*) Representative images of coronal brain sections from control (*Foxf2*^fl/fl^) and *Foxf2*-CKO mice subjected to 2,3,5-Triphenyltetrazolium Chloride (TTC) following MCAO and the relative infarct volume are shown; n = 6 mice per group. (*I*) Statistical analysis of the survival rate of the *Foxf2*-CKO mice after MCAO. (*J*–*L*) OFT, rotarod test and mNSS test of control (*Foxf2*^fl/fl^) and *Foxf2*-CKO mice after MCAO; n = 8. (*M* and *N*) The expression levels of vascular permeability-related proteins were compared between the control (*Foxf2*^fl/fl^) and *Foxf2*-CKO mice after MCAO, n = 3. Data are shown as mean ± SEM. Significance was calculated using paired two-tailed Student’s *t* test. *P < 0.05, **P < 0.01, ***P < 0.001, ns, not significant.

Therefore, we proceeded with functional studies using *Gfap^c^*^re^; *Foxf2*^fl/fl^ mice [*Foxf2-*conditional knockout (CKO) mice]. In Foxf2-CKO mice subjected to MCAO, immunolabeling of the V-SVZ region revealed an unchanged density of GFAP^+^ cells but a 90% reduction in GFAP^+^ FOXF2^+^ dual-positive cells, demonstrating efficient *Foxf2* ablation specifically within the target ependymal cell subpopulation (Fig. 5 *A* and *B*). In addition, *Foxf2*-CKO mice presented significantly increased vascular leakage (Fig. 5 *C* and *D*) and markedly reduced cerebral blood flow (Fig. 5 *E* and *F*). Furthermore, these mice had larger infarct volumes (Fig. 5 *G* and *H*) and higher mortality rates (Fig. 5*I*). Functional neurological deficits were assessed via the modified neurological severity score (mNSS), the open field test (OFT), and the rotarod test (37, 38). Compared with the control mice, the *Foxf2*-CKO mice traveled shorter distances, spent less time on the rotarod, and had higher neurological severity scores, indicating more severe neurological damage (Fig. 5 *J–L*). These findings highlight the crucial role of GFAP^+^ FOXF2^+^ EP in repairing BBB damage; their absence exacerbates BBB dysfunction and increases neurological impairment.

To investigate whether changes in vascular integrity correlate with alterations in the expression of key proteins, we performed western blot analysis on a panel of proteins associated with cell–cell junctions. Our findings revealed that the expression levels of tight junction (TJ) proteins, such as ZO1 and OCCLUDIN, remained largely constant. In contrast, the expression of adherens junction proteins, including CD31 and VE-cadherin (CDH5) (39), was significantly reduced in the *Foxf2*-CKO mouse group. Additionally, the expression of markers of PC and smooth muscle cells, specifically PDGFRB and α-SMA, was also significantly decreased in *Foxf2*-CKO mice (Fig. 5 *M* and *N*). These results suggest that the number of GFAP^+^ FOXF2^+^ EP does not influence development but has a significant effect on BBB leakage during aging.

To inhibit the expression of *Foxf2* in *Gfap^+^* EP, we employed the GfaABC1D promoter in conjunction with local adeno-associated virus (AAV) 2/5-mediated delivery of three shRNAs targeting *Foxf2* in *Gfap*^+^ EP (*SI Appendix*, Fig. S9*A*). Physiologically, we confirmed that the weight gain process and vascular permeability of the mice did not significantly change after virus injection (*SI Appendix*, Fig. S9 *B–D*). Following stroke, the Foxf2 shRNA group showed significantly increased vascular leakage of the 250 kDa tracer (*SI Appendix*, Fig. S9 *E* and *F*), along with reduced cerebral blood flow, expanded infarct volume, and impaired motor function.(*SI Appendix*, Fig. S9 *G–M*). These findings suggest that the inhibition of *Foxf2* in *Gfap*^+^ EP exacerbates BBB damage and impairs neural function.

To evaluate off-target effects of viral infection, we performed immunofluorescence staining on SVZ sections at multiple anatomical levels, focusing on ependymal cell infection. As the distance from the injection site increased, the number of infected EP progressively decreased, indicating spatial restriction of viral infection. Notably, GFAP^−^mCherry^+^ cells were rarely observed at any level examined, suggesting limited nonspecific infection (*SI Appendix*, Fig. S10 *A–C*).

### Overexpression of the Foxf2 Gene in GFAP-Positive Cells Restores BBB Damage after Stroke.

**Fig. 6.**
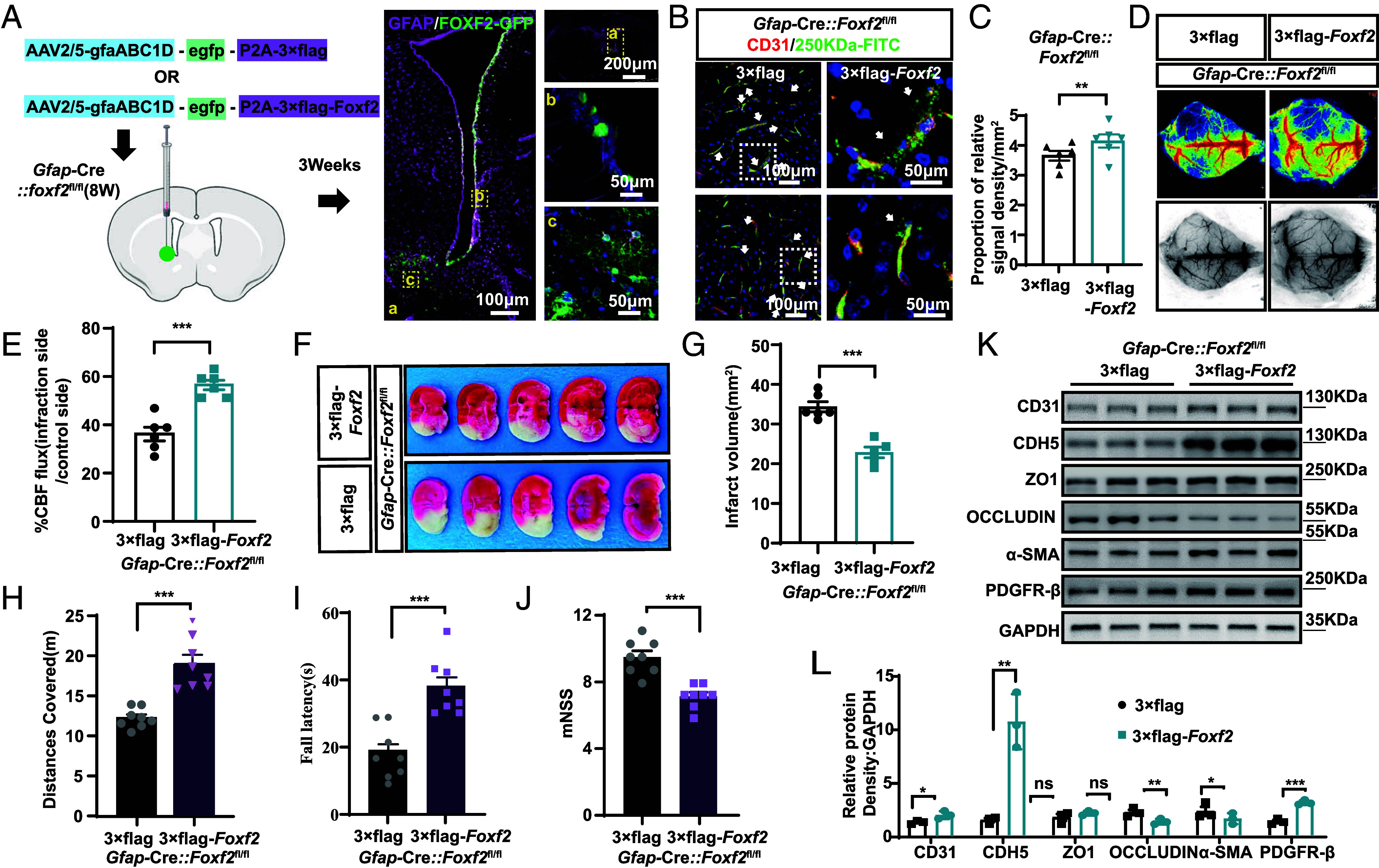
Overexpression of *Foxf2* in ependymal GFAP-positive cells restores BBB integrity after stroke. (*A*) Description of the *Foxf2*-overexpressing AAV injection protocol. (*B* and *C*) Quantitative comparison of 250 kDa FITC and CD31 costaining in Foxf2-CKO vs. Foxf2-overexpressing mice after I/R. (*D*) Representative images and quantification of laser Doppler measurements of blood flow signals in *Foxf2*-CKO mice and *Foxf2-*overexpressing mice following I/R, n = 6 each. (*E*) Quantitative analysis of the data in (*D*). (*F*) Representative images of *Foxf2*-CKO mice and *Foxf2-*overexpressing mice subjected to TTC following I/R and the relative infarct volume are shown; n = 6 mice per group. (*G*) Quantitative analysis of the data in (*F*). (*H*–*J*) OFT, rotarod test and mNSS test of *Foxf2*-CKO mice and *Foxf2-*overexpressing mice after MCAO, n = 8. (*K* and *L*) Comparison of the expression levels of vascular permeability-related proteins between the *Foxf2*-CKO and *Foxf2*-overexpressing mice, n = 3 each. Data are shown as mean ± SEM. Significance was calculated using paired two-tailed Student’s *t* test. *P < 0.05, **P < 0.01, ***P < 0.001, ns, not significant.

To investigate whether targeting GFAP^+^ ependymal FOXF2 cells restore BBB integrity after stroke, we engineered a recombinant AAV serotype 2/5 (AAV2/5) carrying mouse Foxf2 cDNA (NM_010225) under transcriptional control of the GfaABC1D promoter. This vector, designated AAV-GfaABC1D::*Foxf2*, was constructed specifically for targeted gene delivery to GFAP^+^ EP. 3 wk after intracerebroventricular AAV injection, GFP reporter fluorescence showed focal enrichment at the injection tract, with predominant localization to the V-SVZ lateral wall (Fig. 6*A*), confirming efficient and region-restricted ependymal transduction. We subsequently validated the therapeutic potential of *Foxf2* overexpression. In *Foxf2*-CKO mice, the targeted restoration of *Foxf2* significantly reduced vascular leakage, improved cerebral perfusion, and diminished infarct volume (Fig. 6 *B–G*). Furthermore, *Foxf2* overexpression significantly improved neurological function in *Foxf2*-CKO mice, rescuing deficits in behavioral scores and motor performance (Fig. 6 *H–J*). Notably, *Foxf2* overexpression selectively upregulated PDGFRβ and CDH5 (Fig. 6 *K* and *L*), suggesting its potential role in promoting processes critical for vascular stabilization.

### GFAP^+^ FOXF2^+^ EP Are Dependent on the DLL4–NOTCH Pathway to Regulate Vascular Permeability.

**Fig. 7.**
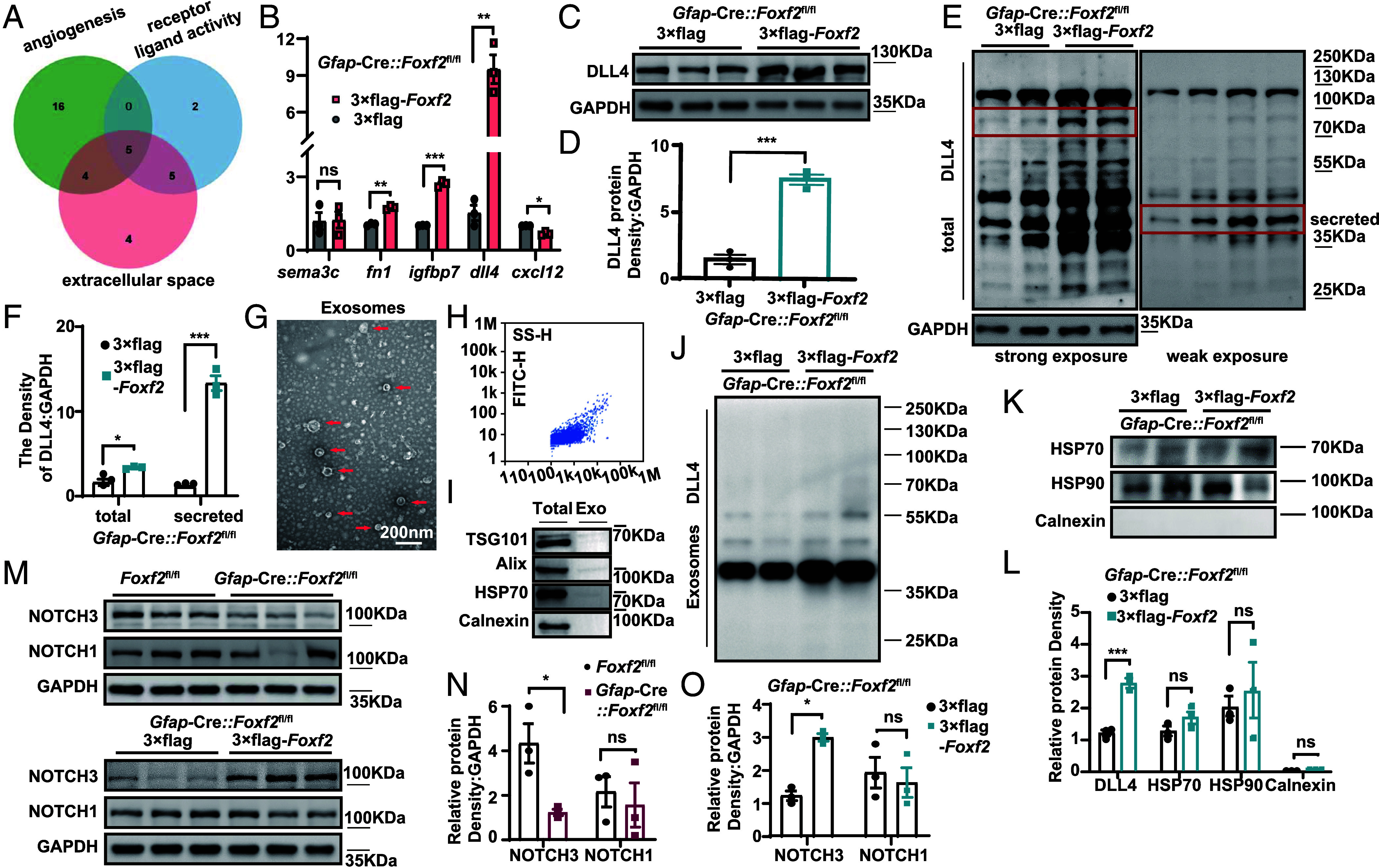
FOXF2 promotes BBB repair in GFAP-positive cells by increasing DLL4 secretion. (*A*) Venn diagram displaying the key molecules in angiogenesis-regulating pathways from the GO functional analysis cluster. (*B*) The five key angiogenesis-regulating molecules depicted in the Venn diagram were validated by real-time PCR in vivo. (*C* and *D*) Quantitative analysis of DLL4 expression in Foxf2-CKO mice injected with Foxf2-overexpressing AAV (n = 3 per group). (*E* and *F*) Western blotting was used to quantitatively evaluate the total protein and secreted protein expression levels of DLL4 in *Foxf2*-CKO mice and *Foxf2-*overexpressing mice. (*G*–*I*) Western blotting, electron microscopy, and nanoparticle tracking analysis were used to detect the purity of extracellular vesicles isolated from the brain tissues of *Foxf2*-CKO mice and *Foxf2-*overexpressing mice. (*J*–*L*) Quantitative comparison of DLL4 expression in brain tissue exosomes between *Foxf2*-CKO mice and *Foxf2-*overexpressing mice. (*M*–*O*) Western blot analysis of the key downstream molecule Notch in *Foxf2*-CKO mice and *Foxf2-*overexpressing mice; n = 3 each. Data are shown as mean ± SEM. Significance was calculated using paired two-tailed Student’s *t* test. *P < 0.05, **P < 0.01, ***P < 0.001, ns, not significant.

On the basis of our previous BP analysis, confirming that these cells are involved in angiogenesis-related functions (Fig. 2*F*), we further conducted Gene Ontology (GO) analyses focused on the Molecular Function (MF) and Cellular Component (CC) categories. Specifically, MF terms related to receptor–ligand activity and CC terms associated with extracellular components were selected. By intersecting gene sets from all three GO categories, BP, MF, and CC, we identified five candidate extracellular molecules that may mediate vascular leakage through receptor–ligand interactions (Fig. 7*A*). Next, we validated the expression levels of these five molecules in the infarcted tissue of *Foxf2-*overexpressing mice via real-time PCR. Among them, *Dll4* showed the most pronounced upregulation (Fig. 7*B*), suggesting that FOXF2 may promote BBB repair primarily through *Dll4*. Our results demonstrated that *Foxf2* overexpression directly induced a significant increase in DLL4 expression (Fig. 7 *C* and *D*). Moreover, the DLL4–NOTCH pathway operates through a well-defined canonical signal transduction cascade, with clear upstream ligands and downstream effectors, serving as definitive regulators of vascular integrity. Therefore, we prioritized DLL4 for subsequent research because of its established signaling framework and the tractability of target validation.

To explore how DLL4 might exert its effects, we considered its potential forms of intercellular communication. As a membrane-bound ligand, DLL4 can undergo proteolytic cleavage to generate a secreted fragment, and can also be packaged into exosomes for extracellular transport (40, 41). Therefore, we examined the changes in the expression of secreted DLL4, which has a relatively small molecular weight because it contains only the extracellular sequence (41). In *Foxf2*-overexpressing mice subjected to MCAO, both the full-length DLL4 protein (~75 kDa) and its secreted fragment (~40 kDa) were significantly upregulated, as confirmed by quantitative western blot analysis of brain lysates (Fig. 7 *E* and *F*). Then, we isolated exosomes from *Foxf2*-overexpressing mice and control mice after MCAO (Fig. 7 *G–I*). *Foxf2* overexpression specifically increased the exosomal DLL4 content without altering the global exosome abundance, as evidenced by comparable levels of constitutive exosome biomarkers (HSP90 and HSP70) (Fig. 7 *I–L*). This finding confirms that *Foxf2* overexpression in GFAP-positive cells can promote DLL4 secretion in exosomes.

In addition, in *Foxf2*-CKO mice, the expression of NOTCH1 did not significantly change, whereas the expression of NOTCH3 was significantly reduced. Similarly, in the *Foxf2-*overexpressing mouse group, the changes in NOTCH1 expression were not significant, whereas NOTCH3 expression increased significantly (Fig. 7 *M–O*). Unlike other NOTCHs, NOTCH3 is expressed primarily in PC and smooth muscle cells. NOTCH3 is important for the survival of PC and smooth muscle cells and plays a critical role in smooth muscle cell blood vessel integrity and BBB function in the mammalian vasculature (42). These findings imply that FOXF2 may modulate BBB permeability by modulating the DLL4–NOTCH signaling pathway.

To definitively establish whether the reparative effects elicited by Foxf2 overexpression depend on *Dll4–Notch* signaling, we crossed Foxf2-CKO mice with *Dll4* ^fl/fl^ mice, generating *Dll4* and *Foxf2* double-gene knockout mice (*SI Appendix*, Fig. S11*A*). Conversely, *Foxf2* overexpression in double-knockout mice did not significantly reduce BBB permeability (*SI Appendix*, Fig. S11 *B–G*), improved neurological function (*SI Appendix*, Fig. S11 *H–J*), and did not alter the expression of vascular integrity-related proteins (*SI Appendix*, Fig. S11 *K–M*). These results suggest that *Foxf2* expression is dependent on *Dll4* to regulate BBB permeability.

To explore how cytoplasmic FOXF2 regulates DLL4, distinct from its nuclear role, we used immunoprecipitation and proteomics to identify FOXF2-interacting proteins in vitro with *Foxf2* overexpression. (*SI Appendix*, Fig. S12 *A–C*) Mass spectrometry revealed 20 potential binding partners, including CD44, which regulates extracellular signal-regulated kinase (ERK) phosphorylation and DLL4 expression (*SI Appendix*, Fig. S12 *D* and *E*). According to published literature (43, 44), the intracellular segment of the transmembrane protein CD44, through tyrosine-protein kinase (Src), regulates the protein ERK, which is subsequently phosphorylated in the nucleus, thereby regulating the expression of multiple downstream genes, including *Dll4* (45). FOXF2 bound CD44 in vitro (*SI Appendix*, Fig. S12*F*) and colocalized with CD44 in the V-SVZ in vivo (*SI Appendix*, Fig. S12 *G* and *H*). In *Foxf2*-overexpressing MCAO mice, DLL4 expression and ERK2 phosphorylation were significantly increased (*SI Appendix*, Fig. S12 *I* and *J*). In contrast, Foxf2-CKO mice showed a marked reduction in DLL4 expression, with only a slight decrease in ERK2 phosphorylation (*SI Appendix*, Fig. S12 *K* and *L*).

## Discussion

Our study provides a comprehensive examination of the molecular and functional diversity of EP within the SVZ following neural injury, with a particular focus on the role of GFAP^+^ FOXF2^+^ EP in BBB repair. While the SVZ has long been recognized for its involvement in neural regeneration, the heterogeneity of its EP has been revealed through single-cell RNA sequencing in recent studies (9, 10, 46). However, the specific functional roles of these heterogeneous ependymal cell populations following neural injury remain largely uncharacterized. Our single-cell RNA sequencing analysis revealed that GFAP^+^ FOXF2^+^ EP exhibit sustained upregulation of expression during the early stages poststroke, implicating them in the neural injury response.

Previous research has indicated that GFAP^+^ EP are highly expressed following neural injury (47–49), suggesting their potential involvement in repair processes. However, the specific roles of these cells have remained largely unclear. In this study, we demonstrated that GFAP^+^ FOXF2^+^ EP, a previously unrecognized subset, play a crucial role in promoting BBB repair. Deletion of these cells in knockout mice resulted in exacerbated BBB damage, providing direct evidence of their essential role in maintaining BBB integrity after injury. In addition, we propose that these GFAP^+^ FOXF2^+^ cells, which we identified as E1-type EP on the basis of the comparison of biomarkers and spatial distribution characteristics, may influence BBB repair indirectly through exosome-mediated signaling. This finding complements previous findings that E1-type EP function primarily to regulate CSF flow and contribute to metabolic waste clearance.

At the molecular level, we detected elevated expression of DLL4, a key Notch ligand that regulates endothelial function, in a subset of GFAP^+^ FOXF2^+^ EP. Notably, FOXF2 exhibited ectopic cytoplasmic localization in these cells, in contrast to its typical nuclear expression in EC and PC (25). Similar ectopic expression of OLIG2, a canonical oligodendrocyte transcription factor, has also been reported specifically in EP, further supporting the notion of transcriptional plasticity in the SVZ niche. Our subsequent experiments confirmed that GFAP^+^ FOXF2^+^ EP release DLL4 in exosomal vesicles. DLL4 is a transmembrane ligand for Notch receptors and is highly expressed in EC during development, where it regulates critical processes such as endothelial sprouting and arterial specification (50). Although DLL4 expression is rare in adults, it is reactivated after vascular injury and is not restricted to EC (40, 51). Recent studies have demonstrated that soluble DLL4, comprising only its extracellular portion, can activate Notch signaling in EC, leading to the upregulation of vascular-endothelial cadherin but not TJ proteins such as ZO1 (52). Together, these findings highlight the importance of DLL4 in BBB repair and connect FOXF2 to DLL4-mediated Notch signaling, while illustrating the injury-induced plasticity of ependyma-derived factors.

A key unresolved question remains as to how this spatially limited population of resident EP could contribute to brain-wide BBB restoration. We hypothesize that activation of the DLL4–Notch signaling axis, which may be mediated through exosomal secretion, represents a plausible contributing mechanism. However, this proposed pathway alone is unlikely to fully account for the observed widespread effects. The precise spatiotemporal dynamics, including how the initial signal might be amplified or relayed across distinct brain regions, are not yet defined. Additionally, while DLL4 secretion correlates with BBB restoration, the detailed mechanistic axis remains to be fully elucidated, including whether endothelial NOTCH signaling is the direct target and the spatial distributions of DLL4 and NOTCH activity in the injured brain.

Furthermore, several methodological limitations of this study should be noted. First, the direct evidence demonstrating the migration of exosomes from the SVZ to distal lesion sites is currently lacking, and the existing literature does not yet provide conclusive support for such long-range signaling in this specific context. Thus, how exosomal Dll4 contributes to BBB repair in the infarct region remains an open question for future investigation. Second, as noted in a prior review (3), the genetic tools used for ependymal targeting may lack specificity, with potential off-target effects on neighboring cell populations. This makes it difficult to conclusively attribute the observed phenotypes or protein functions solely to EP. Future studies should therefore employ more precise targeting strategies to delineate cell-autonomous functions and directly trace exosome trafficking to determine their contribution to the observed restorative outcomes.

In summary, we identified a distinct subset of EP characterized by coexpression of GFAP and FOXF2 that emerges after neural injury and appears to contribute to BBB repair. These cells were found to secrete DLL4, which is associated with BBB restoration and represents a potential mediator of this process. While the spatial reach and detailed molecular mechanisms of this influence require further clarification, our findings establish a cellular and molecular framework for understanding endogenous mechanisms of BBB repair.

## Materials and Methods

*Foxf2*^fl/fl^ mice, *Dll4*^fl/fl^ mice, and *Gfap*-Cre mice originated from Cyagen Company (Suzhou, China). *Gfap*-Cre::*Foxf2*^fl/fl^ and *Gfap*-Cre::*Foxf2*^fl/fl^;*Dll4*^fl/fl^ mice were obtained through breeding. 5 × FAD mice (Transgenic Mice with Five Familial Alzheimer’s Disease Mutations) was purchased from Jackson Laboratory.The detailed procedures for establishing the MCAO and photochemical thrombosis models, along with full experimental details including protocols, key reagents, and resources, are provided in *SI Appendix*, *Materials and Methods*.

## Supplementary Material

Appendix 01 (PDF)

Dataset S01 (XLSX)

Dataset S02 (XLSX)

Dataset S03 (XLSX)

Dataset S04 (XLSX)

## Data Availability

Data generated or analyzed during this study are included in *SI Appendix*, *Materials and Methods*. The accession number for the single cell RNA-seq datasets (PRJNA201322549) (53) and the processed data can be found in *SI Appendix*, Tables S1–S4. Refs. 54–56 provide the raw data for refs. 28–30, respectively. The results of the comparative analysis between these datasets and our own sequencing data are shown in *SI Appendix*, Fig. S3. All other data are included in the manuscript and/or supporting information.
