# Supplementary material for: GFAP+ FOXF2+ ependymal cells promote blood–brain barrier repair via DLL4–NOTCH signaling after neural injury
Source: Proc Natl Acad Sci U S A. 2026 Mar 24;123(13):e2520352123. doi: 10.1073/pnas.2520352123 (PMC13037844; doi:10.1073/pnas.2520352123)
Supplement: Supplementary file 1 — Appendix 01 (PDF) [file pnas.2520352123.sapp.pdf]

Supplementary Information for

GFAP+ FOXF2+ Ependymal Cells Promote Blood-Brain Barrier Repair via DLL4-NOTCH Signaling after Neural Injury

Qi Xie et al.
Corresponding author: yangqwmlys@tmmu.edu.cn (Q.W.)

The PDF file includes:

Supplementary methods
SI References
Figs. S1 to S12

Other Supplementary Material for this manuscript includes the following:

Legends for Datasets S1 to S4
Dataset S1 Single-cell sequencing clustering of the SVZ region
Dataset S2 Subpopulations of ependymal cells further classified from single-cell sequencing of the SVZ
Dataset S3 Single-cell sequencing clustering of the infarcted region
Dataset S4 Subpopulations of ependymal cells further classified from single-cell sequencing of the infarct region

Materials and methods

Key resources table

REAGENT or RESOURCE	SOURCE	IDENTIFIER
Antibodies		
Goat anti-CD31	R&D Systems	AF3628
Goat anti-IBA1	Abcam	ab5076
Goat anti-GFAP	Abcam	ab53554
Goat anti-BLBP	Abcam	Ab32432
Donkey anti-Goat Secondary Antibody, AF Plus 488	Thermo Fisher	A32814
Donkey anti-Goat Secondary Antibody, AF Plus 647	Thermo Fisher	A21447
Donkey anti-Rabbit Secondary Antibody, AF Plus 555	Thermo Fisher	A31572
Donkey anti-Mouse Secondary Antibody, AF Plus 555	Thermo Fisher	A32773
Donkey anti-Rabbit Secondary Antibody, AF Plus 647	Thermo Fisher	A31573
Donkey anti-Rat Secondary Antibody, AF Plus 488	Thermo Fisher	A21208
Donkey anti-Mouse Secondary Antibody, AF Plus 488	Thermo Fisher	A21202
Rat anti-CD44	Santa Cruz	SC18849
Mouse anti-FOXJ1	Thermo Fisher	14-9965-80
Rat anti-CD144(VE-cadherin)	Thermo Fisher	14-1441-82
Mouse anti-FOXF2	Santa Cruz	SC101043
Rabbit anti-KI67	CST	D3B5
Rabbit anti-β-Amyloid	CST	D5402
Rabbit anti-ZO1	Affinity Biosciences	AF5145
Rabbit anti-PDGFRβ	CST	3169S
Rabbit anti-β-catenin	CST	8480T
Mouse anti-α-SMA	R&D Systems	MAB1420
Rabbit anti-α-SMA	Abcam	ab5694
Goat anti-DLL4	R&D Systems	AF1389
Rabbit anti-CD44	Abcam	ab189524
Rabbit anti-NOTCH1	Abcam	ab52627
Rabbit anti-VE Cadherin	Abcam	ab33168
Bacterial and virus strains		
pAAV-GfaABC1D-egfp-P2A-3xflag-WPRE	OBIO	H6019
pAAV-GfaABC1D-egfp-P2A-*Foxf2*-3xflag-WPRE	This study	N/A
PAAV-GfaABC1D-mCherry-3xflag-miR30ShRNA(NC)-WPRE	OBIO	Y6432
pAAV-GfaABC1D-mCherry-3xflag-miR30shRNA(*Foxf2*-1)-WPRE	This study	N/A
pAAV-GfaABC1D-mCherry-3xflag-miR30shRNA(*Foxf2*-2)-WPRE	This study	N/A
pAAV-GfaABC1D-mCherry-3xflag-miR30shRNA(*Foxf2*-3)-WPRE	This study	N/A
Biological samples		
None		

Chemicals, peptides, and recombinant proteins		
PageRuler Plus Prestained Protein Ladder	Thermo Fisher	26619
Critical commercial assays		
None		
Deposited data		
Mouse single-cell RNA-seq dataset of the infarct side	This study	N/A
Mouse single-cell RNA-seq dataset of the SVZ	This study	N/A
Transcriptome of *Foxf2* overexpression	This study	N/A
Interaction proteomics of FOXF2 overexpression	This study	N/A
Experimental models: Cell lines		
Primary astrocyte cells cultures	This study	N/A
Experimental models: Organisms/strains		
Foxf2 $^{fl/fl}$ mice	Cyagen	S-CKO-02446
Gfap-Cre mice	Cyagen	C001062
Dll4$^{fl/fl}$ mice	Cyagen	S-CKO-11772
Gfap-Cre::*Foxf2*$^{fl/fl}$ and *Dll4*$^{fl/fl}$ mice	This study	N/A
Gfap-Cre::*Foxf2*$^{fl/fl}$ mice	This study	N/A
5×FAD	Jackson Laboratory	008730
Recombinant DNA		
None		
Software and algorithms		
CellSens Demention software	Olympus	http://www.olympus-lifescience.com/en/
GraphPad Prism 8.02	GraphPad Software	http://www.graphpad.com
ImageJ	NIH	https://imagej
SPSS 22.0	IBM	https://www.ibm.com/analytics/spss
Other		
None		

EXPERIMENTAL MODEL AND SUBJECT DETAILS

Animals

Gfap-Cre:: *Foxf2*$^{fl/fl}$

Conditional *Foxf2*$^{fl/fl}$ mice were created by inserting loxp flanking exon 1, which then removed the start codon as well as part of the coding region from the *Foxf2* gene after *Gfap*-Cre mediated recombination. *Foxf2*$^{fl/fl}$ mice and *Gfap*-Cre (Product Number：C001062) originated from Cyagen Company (Suzhou, China).

Gfap-Cre::*Foxf2*$^{fl/fl}$;*Dll4*$^{fl/fl}$

Conditional *Dll4*$^{fl/fl}$ mice were created by insertion of one loxp cassette upstream of exon 3 and a second one downstream of exon 6. Cre expression will remove exon 3~6 and flanking intronic sequence including the splice acceptor and donor and causes amino acid changes and early truncation. *Gfap*-Cre::*Foxf2*$^{fl/fl}$ and *Dll4*$^{fl/fl}$ mice were bred together to generate double transgenic mice *Gfap*-cre:: *Foxf2*$^{fl/fl}$;*Dll4*$^{fl/fl}$.

5×FAD

5×FAD (Jax:008730) originated from The Jackson Laboratory.

The mice used in the experiment were C57BL/6J background and had been backcrossed for at least six generations. During the feeding process, all mice (2-5 per cage) were kept in a constant temperature environment of 20-25 °C, with a 12/12 hour light/dark cycle, 30-70% humidity, and provided with sufficient food and water. Genotyping of offspring mice by alkaline lysis method (1). All animal experiments have been approved by the Experimental Animal Welfare and Ethics Committee of the Third Military Medical University.

Mouse Neural Injury Model

Transient focal cerebral ischemia model

As mentioned earlier, the transient focal cerebral ischemia model in mice is achieved by occluding the lumen of the left middle cerebral artery (tMCAO) with a suture (2). In short, the mice were grouped and modeled using a double-blind method. According to the completely randomized design (CRD) scheme, animals were divided into an experimental group and a control group, with the experimental group consisting of male mice from the same litter. Anesthetize mice with isoflurane (2-3% induction, 1-1.5% maintenance) and then expose the unilateral common carotid artery (CCA), external carotid artery (ECA), and internal carotid artery (ICA) through a skin incision. Apply microvascular aneurysm clamp to CCA and ICA for clamping. Insert a 2cm long round head nylon suture (Jialing, Shanghai, China) into the arterial incision of the ECA and push it towards the distal end to a predetermined distance of 8-9mm from the carotid bifurcation to occlude the MCA. After 90 minutes of focal cerebral ischemia, gently remove the suture plug, tighten the neck loop suture at the bottom of the ECA stump, undo the CCA, suture the skin incision, and stop anesthesia. After the experiment is completed, a blood flow meter can be used to detect blood flow in mice to evaluate the surgical results. We only keep mice with cerebral blood flow (CBF)<15%. Except for arterial occlusion, sham operated mice underwent the same surgical procedure as described above(2).

Photochemical thrombosis model

Prepare a 10 mg/mL physiological saline solution of rose red (Sigma) in advance, and inject 0.1 mL via intraperitoneal or orbital vein injection. After 5 minutes, induce anesthesia in mice with isoflurane and fix them in a stereotactic frame. During the experiment, a 4.5mm aperture cold light source fiber bundle was used. A window of appropriate size was polished on the mouse skull according to the aperture of the fiber bundle, and the irradiation was maintained for 15 minutes. Subsequently, the scalp was sutured and the mice were placed on a heating pad to maintain body temperature and wait for awakening.(3).

Single-cell RNA-seq and analysis

Separate SVZ area

According to the established protocol(4), after perfusion with cold sterile PBS, mouse brain tissue was peeled off, and fresh mouse brain tissue was placed in complete culture medium for separation in ice . Firstly, carefully divide the brain tissue into two parts along the sagittal plane, and then use fine forceps to precisely remove the area from the hippocampus to the cerebellum. Gently lift up the cortex and use a microsurgical knife (Rayward) to carefully remove the corpus callosum beneath the cortex, exposing the SVZ outer wall (LV) brain. Subsequently, remove the midbrain and striatum beneath the LV. Then carefully remove a piece of SVZ outer wall with a thickness of about 50 μ m under high magnification using a microsurgical knife, and continue to prepare single-cell suspension according to the instructions (Meitianli 130-107-677). Subsequently, single-cell transcriptome analysis will be conducted to characterize the cellular diversity within that specific ecological niche.

Single cell preparation and library construction

For the Rhapsody experiment, the whole process was done by following manufacturer's (BD Biosciences) protocol(5). Collect the infarcted hemisphere of mice after perfusion with cold and sterile PBS, and prepare single-cell suspension according to the instructions (130-107-677 miltenyi biotec). After detecting cell activity (Calcein AM BD, DRAQ7 TM BD), prepare a single-cell dilution according to cell concentration and load 20000 cells into Cartridge (BD). According to BD Rhapsody ™ The reverse transcriptome kit and BD whole transcriptome (WTA) amplification kit were used to process the samples. After the construction of the library, Qubit 2.0 was used for preliminary quantification, then agarose gel electrophoresis and Agilent 2100 were used to detect the library fragments, and qPCR method was used to accurately quantify the effective concentration of the library to ensure the library quality. After passing the library inspection, Illumina NovaSeq 6000 sequencing was performed based on the effective concentration of the library and the data production requirements, with a sequencing strategy of PE150.

Single-cell RNA-seq analysis

Single-cell RNA-sequencing data were processed as we previously reported(2). Each sample was aligned to the mouse reference genome (mm10) and raw expression data were analyzed by R (version 3.5.1). Then, cells of all of the datasets were analyzed for their unique molecular identifier (UMI) and mitochondrial gene counts. Low quality cells (<3 cells/gene or <200 genes/cell) were excluded from the overall experiment. Data were integrated in a standardized workflow, as recommended by the developers of the "Seurat" R package(6).

The initial resolution value employed for the classification and analysis of cell types in the infarct periphery was 0.1, allowing for comprehensive and rapid assessment of the change profiles of various nerve cells following injury. In contrast, a resolution value of 0.8 was employed in the svz group with the aim of classifying the cells in greater detail in order to identify more specific subclasses with a reduced number of cells. The cell type annotations were referenced to highly expressed signature genes available online at the CellMarker database(7) and the Seurat package FindAllMarkers program, as well as to cellular markers reported in the literature for neuronal cells and the SVZ region(8). Gene ontology (GO) networks based on DEGs were created using the Functional Annotation Bioinformatics Microarray Analysis (DAVID)(9).

To characterise the transdifferentiation subpopulations of each cell subsets in the SVZ region, monocle analysis was utilised to evaluate cell trajectories as the previously reported(10).

The differentially expressed genes were identified for trajectory study by comparing the transcriptomes among all cells in the SVZ region. Then, we used monocle to construct lineage trajectory and branch points by analyzing the differentially expressed genes. Gene expression heatmap is based on lineage trajectory and performed by monocle.

Cerebral blood flow (CBF) measurements

As mentioned earlier, we used a laser speckle imaging device (RWD Shenzhen, China) to monitor the CBF status of the cortex (2). In short, the charge coupled device camera was placed above the head of anesthetized mice and the entire skull surface was irradiated with a laser diode (785nm) to allow for continued penetration of the brain in a diffuse manner. Measurements were taken on both hemispheres of the brain 15 minutes before transient middle cerebral artery occlusion (tMCAO) to obtain baseline blood flow values in mice, followed by continuous monitoring throughout the experiment until 15 minutes after reperfusion. We only included mice that showed a CBF reduction of at least 75% compared to baseline in the subsequent experimental analysis.

Evans blue assay

The Evans blue assay was performed as previously described(11). Prepare Evans Blue solution (30 mg/mL, dissolved in 0.9% physiological saline) in advance and administer it via the tail vein at a dose of 4 mL/kg. After 24 hours, the mice were perfused with 60 mL of cold PBS, and then the mouse brain tissue was sliced and photographed using a mold. After completion, homogenize the same level tissue blocks from different groups to obtain protein solution. Then centrifuge at 10000 x g for 30 minutes at 4°C. Transfer 0.2mL of the supernatant to a 96 well plate and measure the concentration of Evans blue in the supernatant at 610nm using a spectrophotometer.

Dextran tracer assay

As described in detail in the previous study(12) prepare a suitable concentration of glucan tracer (2 mg/ml Sigma) in advance. Inject 0.1ml through the tail vein or orbital vein, anesthetize to death after 10 minutes, and directly extract the brain tissue. After obtaining the brain tissue, freeze it at -80°C for subsequent frozen section production. The thickness of the sliced brain slices is about 10μm. After drying at room temperature, they are fixed in paraformaldehyde solution for 15 minutes and washed with PBS for subsequent immunofluorescence staining experiments.

Western blot (WB) analysis

We perfused the brain tissue of glass mice with PBS and isolated the cortex, striatum, and subventricular zone of the infarcted hemisphere. We added lysis buffer (60mM Tris HCl (pH 6.8), 5% glycerol, 2% SDS) at a ratio of 1mg/20ul, thoroughly ground it, and collected the supernatant by centrifugation. Then boil at 100°C for 10 minutes. Measure protein concentration using the BCA kit (Beyotime) and add the corresponding volume of loading buffer according to the principle of consistent total protein mass. The protein was separated by 10% SDS-PAGE (Beyotime), and then transferred to polyvinylidene fluoride (PVDF) membrane (sigma). Seal the membrane with Tris buffered saline containing 5% fat free milk (Beyotime) at room temperature for 60 minutes. Then incubate overnight with the first antibody at 4°C. After incubating the secondary antibody conjugated with peroxidase at room temperature for 1 hour, the protein bands were visualized using ECL (invigentech). Then evaluate the optical density using image J.

Immunofluorescence staining

As described in detail in our previous article(13). After perfusion with pre cooled PBS (HyClone), mouse brain samples were fixed with cold 4% paraformaldehyde (Servicebio), dehydrated with 30% sucrose (sangon), and embedded in OCT (sakura). Separate mouse brain tissue into 30μm thick sections in a frozen section machine and store them in cryopreservation solution at -20°C. The slices were blocked with 5% BSA (Beyotime) containing 0.3% Triton - 100(sigma) at room temperature for 1 hour, and then incubated with primary antibody solutions overnight at 4°C.Subsequently, the slices were incubated with the corresponding fluorescent secondary antibodies for two hours. After that, the slices were stained with DAPI (1:5000) (Beyotime) at room temperature for 15 minutes. Finally, images were captured using a confocal fluorescence microscope (Olympus, Japan).

TTC staining

After completing the ischemia-reperfusion model, wait for 24 hours, then anesthetize the mice to death and directly remove the brain tissue. Cut the mouse brain into 7 1mm thick coronal sections using a mold and stain them with 2% TTC (Sangon Biotech) at 37°C. After coloring, transfer the brain slices to 4% paraformaldehyde for fixation (14). Take images with a digital camera and measure them using image analysis software (Adobe Photoshop).

Neurological function assessments

Open field behavioral test

Prior to the test, the mice were numbered using double-blind labeling and placed in the testing room for 24 hours of adaptation before the experiment began. During the formal experiment, mice will be placed in a blue opaque test box (40cm x 40cm x 40mm) for 10 minutes of spontaneous exploration activity. The activity parameters of the mice in the test box will be recorded and collected by a camera connected to a computer. After the test is completed, the number will be matched with the mouse genotype for subsequent grouping analysis. Evaluate the motor ability of mice based on their total distance traveled and exercise time.

Rotarod test

Evaluate the motor function of mice after MCAO model by accelerating the rotation rod test. During the training phase, set the rotation speed to 30rpm/s and force the mice to move on the spinning stick for 5 minutes. If they fall during this period, quickly reset them. Train once a day for 3 days, gradually increasing from 4 rpm/s to 30 rpm at a constant rate on the 4th day and maintaining this speed. The total testing time is 5 minutes, and the time the mice fall is recorded.

mNSS

Multi functional evaluation of nerve damage in mice from aspects such as movement, reflex, sensation, and balance. Including tail lifting test (0-3 points), walking on flat ground test (0-6 points), balance beam test (0-6 points), and reflex deficit test (0-2 points), the higher the score, the more severe the neurological deficit.

Quantitative real-time PCR (qRT-PCR).

Total RNA was extracted using TRI Reagent (sigma), according to the manufacturer's instructions. Add fresh tissue, Trizol, and grinding beads (Servicebio) in a ratio of 50-100mg/ml

for thorough lysis, then, extract and purify RNA according to the instructions. Dissolve the purified RNA precipitate in 20ul EASY Dilution (TaKaRa) and mix well. Then, perform concentration and purity testing using Nano Drop Technologies. Perform reverse transcription and quantitative real-time PCR (qPCR) analysis according to the instructions (PrimeScript ™ RT reagent kit, TaKaRa; SYBR Green, Toyobo).

Isolation and culture of primary astrocyte cells

C57BL/6J neonatal mice born within two days were used in the experiment. Before stripping the brain tissue, the surface was disinfected with 75% alcohol, and then anesthetized with 1% isoflurane (RWD). After removing the dura mater and pia mater, the mouse cortex was peeled off and collected in cold sterile DMEM (1% FBS GBICO) medium. Divide it into tissue blocks of approximately 1mm using sterile surgical scissors, wash with cold PBS, and digest with 0.25% trypsin (1ml/1 brain) (HyClone) in an incubator at 60 rpm and 37°C for 20 minutes. Then, terminate digestion with DMEM (10% FBS). Centrifuge 300g in a centrifuge at 4°C for 10 minutes, then resuspend the cell pellet in DMEM (10% FBS) medium and filter out impurities using a 70μm sieve (biosharp). Spread the collected cells flat in T75 culture bottles (nun), perform half medium exchange after 24 hours, perform full medium exchange after 3 days, and after 7 days, the cells will converge to a density of approximately 85% -90%. Purify the cells (in a 37°C incubator, 250rpm, for 18-24 hours or by colliding the culture bottles with each other).

Brain stereotactic injection

The experiment used 8-week-old C57BL/6J male mice, which were anesthetized with isoflurane (3% for induction and 1.5% for maintenance) and placed on a stereotactic scaffold (RWD). After removing the hair, the mouse skull was exposed, and after localization, a skull drill was used for drilling treatment. In this experiment, adenovirus associated virus (OBIO) was injected. A microsampler (Hamilton) was used to inject 500nl at a rate of 100nL/min. After injection, the needle was left for 5 minutes, and the injection site was determined based on the mouse brain atlas.

Ara-C treatment

This experiment is a 5-day cycle slow administration type, with sterile operation throughout the entire process. According to the administration time and the volume of the osmotic pump, prepare the corresponding concentration of Ara-C (2% sterile physiological saline configuration, Sigma) solution, and inject the Ara-C solution into the micro osmotic pump (RWD, 1001W model); At a flow rate of 0.5 μ L/hour for 5 days, the osmotic pump was connected to a positioning drug delivery needle, and the needle was fixed on the mouse skull with dental cement, located at the anterior fontanelle (1mm lateral midline, 3mm deep brain surface). Then, the drug pump was buried subcutaneously on the back, and after 5 days of infusion, samples were immediately taken for immunofluorescence staining.

Isolation exosomes from mouse brain

Extract Brian exosomes (15) according to the manufacturer's instructions. Briefly, after perfusion and rinsing with PBS buffer, the brain tissue is peeled off. Then gently digest the tissue with the reagents in the kit to release interstitial fluid. Next, filter and centrifuge at low speed three times: 300g for 10 minutes, 2000g for 10 minutes and 10000g for 10 minutes. Subsequently, centrifuge at a speed of 150000g for 2 hours at 4°C. Discard the supernatant and dissolve the precipitate in 0.4ml frozen PBS (containing protease and phosphatase

inhibitors) to obtain small bubbles. After ultracentrifugation, add extracellular vesicles containing 1ml PBS into the ultrafiltration tube. Centrifuge at 4°C and 4000g for 1 minute. Measure the BCA protein concentration of extracellular vesicles after ultrafiltration.

Co-immunoprecipitation and proteomic analysis.

Detailed steps for Co-immunoprecipitation (Co-IP) and proteomic analysis, as well as the mass spectrometry parameters settings, can be found in our previous publication(16). To detect protein interactions, *Foxf2* lentivirus (HANBIO Shanghai) was over expressed into primary astrocytes. First, proteins are extracted from cells expressing FOXF2 using appropriate lysis buffers. Next, affinity purification is conducted to isolate protein complexes that interact with FOXF2. The isolated complexes are thoroughly washed to remove non-specific proteins. Subsequently, the purified complexes are digested into peptides with proteolytic enzymes trypsin (Promega). These peptides are then analyzed by mass spectrometry (MS) to identify and quantify the peptides.

Statistical analysis

The experiment and analysis were conducted without knowing the grouping. The estimation of sample size is based on sample size calculation formula: $n = \frac{(Z_{1-\alpha/2} + Z_{1-\beta})^2\sigma^2}{\delta^2}$ where n represents sample size in each group (assumes sizes of two groups are equal),σ represents standard deviation of the outcome variable,$Z_{1-\alpha/2}$ represents desired level of significance,$Z_{1-\beta}$ represents desired power and δ represents effect size (the difference in means). Statistical analysis Results were expressed as mean±SEM and all statistical parameters and analysis are mentioned in the figure legends respectively. All experiments were performed with randomization of group assignment via number lottery draw, allocation concealment, blinding of operators, blinding of measurements. Data for all experiments were analyzed with Graph Pad Prism 8.0 software.

SI References

1. S. Y. Ren *et al.*, Growth hormone promotes myelin repair after chronic hypoxia via triggering pericyte-dependent angiogenesis. *Neuron* **112**, 2177-2196 e2176 (2024).
2. L. Xie *et al.*, Single-cell RNA sequencing of peripheral blood reveals that monocytes with high cathepsin S expression aggravate cerebral ischemia-reperfusion injury. *Brain Behav Immun* **107**, 330-344 (2023).
3. J. K. Lee *et al.*, Photochemically induced cerebral ischemia in a mouse model. *Surg Neurol* **67**, 620-625; discussion 625 (2007).
4. Z. Mirzadeh, F. T. Merkle, M. Soriano-Navarro, J. M. Garcia-Verdugo, A. Alvarez-Buylla, Neural stem cells confer unique pinwheel architecture to the ventricular surface in neurogenic regions of the adult brain. *Cell Stem Cell* **3**, 265-278 (2008).
5. A. R. Lawrence *et al.*, Microglia maintain structural integrity during fetal brain morphogenesis. *Cell* **187**, 962-980 e919 (2024).
6. T. Stuart *et al.*, Comprehensive Integration of Single-Cell Data. *Cell* **177**, 1888-1902 e1821 (2019).
7. X. Zhang *et al.*, CellMarker: a manually curated resource of cell markers in human and mouse. *Nucleic Acids Res* **47**, D721-D728 (2019).
8. J. P. Magnusson *et al.*, Activation of a neural stem cell transcriptional program in parenchymal astrocytes. *Elife* **9** (2020).

9. W. Huang da, B. T. Sherman, R. A. Lempicki, Systematic and integrative analysis of large gene lists using DAVID bioinformatics resources. *Nat Protoc* **4**, 44-57 (2009).
10. T. Zhao *et al.*, Single-Cell RNA-Seq Reveals Dynamic Early Embryonic-like Programs during Chemical Reprogramming. *Cell Stem Cell* **23**, 31-45 e37 (2018).
11. R. Zhan *et al.*, NAD(+) rescues aging-induced blood-brain barrier damage via the CX43-PARP1 axis. *Neuron* **111**, 3634-3649 e3637 (2023).
12. I. Ozen *et al.*, Loss of Regulator of G-Protein Signaling 5 Leads to Neurovascular Protection in Stroke. *Stroke* **49**, 2182-2190 (2018).
13. X. Y. Xiong *et al.*, Toll-Like Receptor 4/MyD88-Mediated Signaling of Hepcidin Expression Causing Brain Iron Accumulation, Oxidative Injury, and Cognitive Impairment After Intracerebral Hemorrhage. *Circulation* **134**, 1025-1038 (2016).
14. J. Zeng *et al.*, TRIM9-Mediated Resolution of Neuroinflammation Confers Neuroprotection upon Ischemic Stroke in Mice. *Cell Rep* **27**, 549-560 e546 (2019).
15. P. D'Acunzo *et al.*, Isolation of mitochondria-derived mitovesicles and subpopulations of microvesicles and exosomes from brain tissues. *Nat Protoc* **17**, 2517-2549 (2022).
16. Q. Xie *et al.*, Recombinant HNP-1 Produced by Escherichia coli Triggers Bacterial Apoptosis and Exhibits Antibacterial Activity against Drug-Resistant Bacteria. *Microbiol Spectr* **10**, e0086021 (2022).

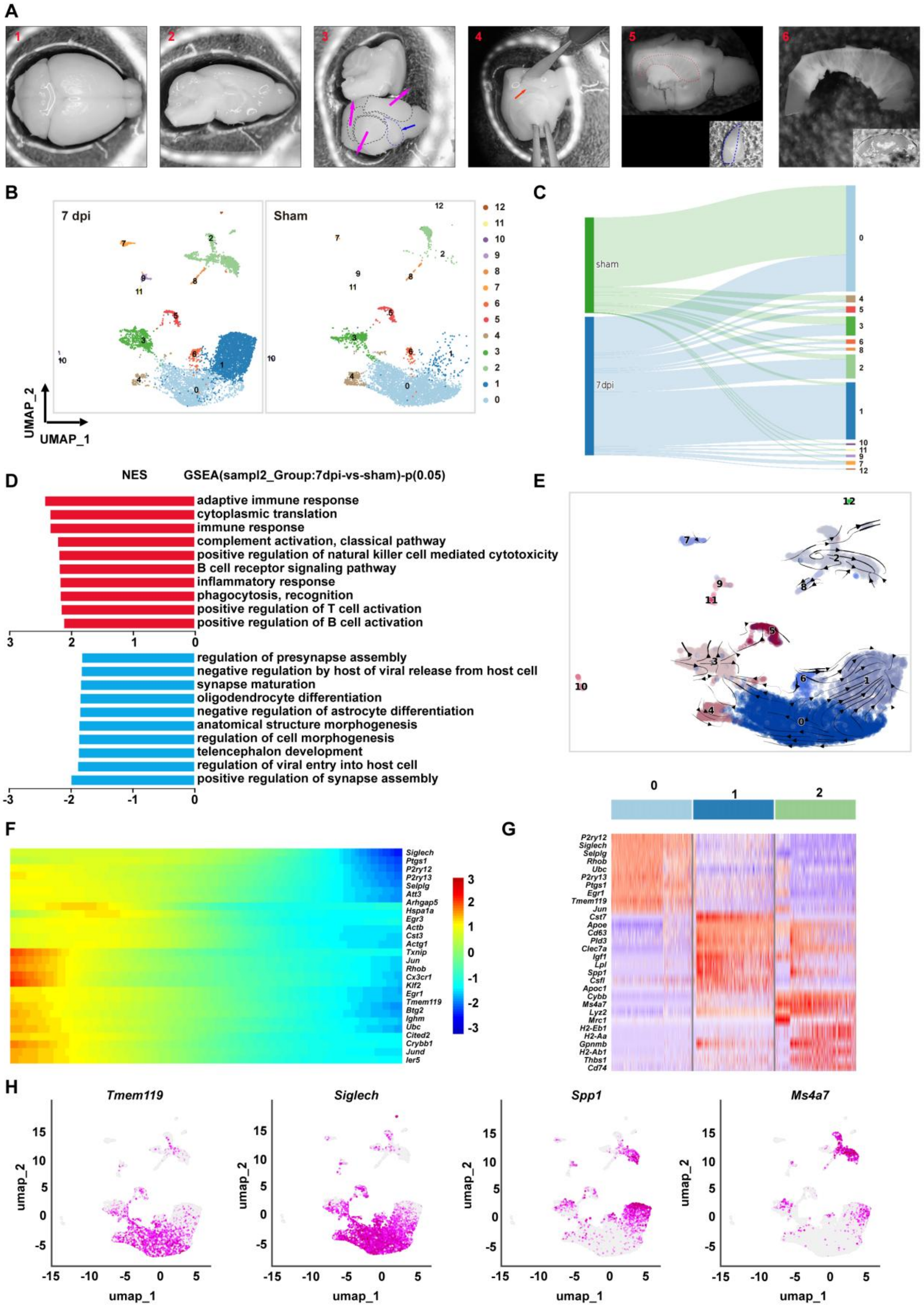

Fig. S1. Transcriptomic characterization of microglia subpopulation

(A) Schematic diagram of left ventricular sidewall anatomy.The black dashed line represents the area to be cut, the purple arrow represents the direction of the incision, the red arrow represents the outer wall (LV wall) of the SVZ area, and the red dashed line represents the LV wall of the entire SVZ area. (B) The microglia subset is further subdivided to characterize the

cell subpopulations. (C) GSEA (Gene Set Enrichment Analysis) analysis comparing the differences in signaling pathways between 7dpi and sham. (D) The Sankey diagram illustrates the differences in both cell numbers and cell proportions between the sham and 7dpi groups for each cell type. (E) RNA velocity analysis is used to assess the dynamic changes in cell states. (F) The pseudotime analysis heatmap shows the regulatory key molecules between cluster 0 and 1. (G) The expression profile heatmap displays the differentially expressed molecules among Cluster 0, 1, and 2. (H) The scatter plot reveals the different marker molecules in cluster 0, 1, and 2.

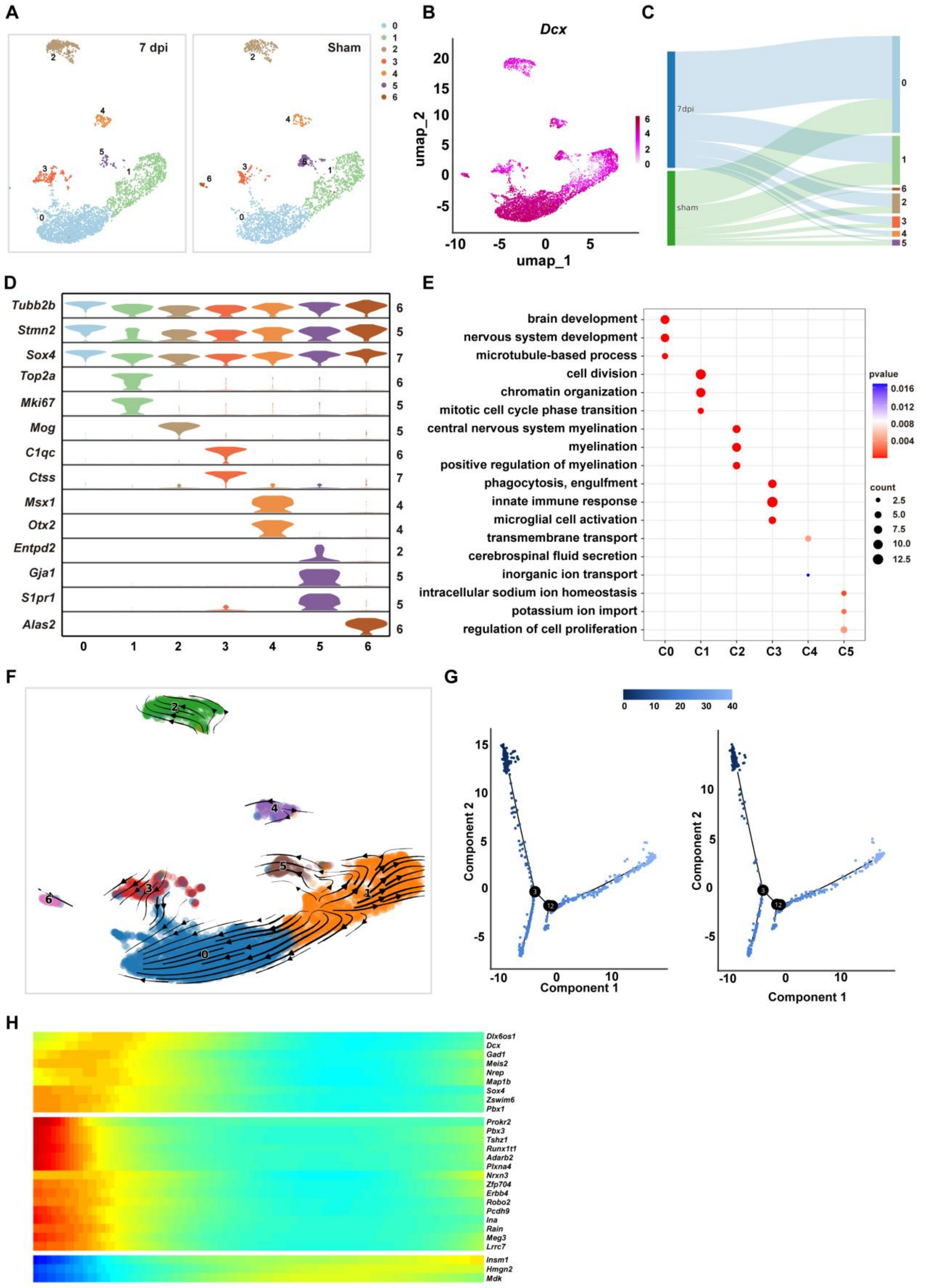

Fig. S2. Transcriptomic characterization of neuroblasts subpopulation

(A) The neuroblasts subset is further subdivided to characterize the cell subpopulations. (B) Scatter plot of the expression distribution of the neuroblast marker gene *Dcx*. (C) The Sankey diagram illustrates the differences in both cell numbers and cell proportions between the sham and 7dpi groups for each cell type. (D) Violin plots of the representative markers for each subpopulation. (E) Functional enrichment analysis of the representative biological processes

for each subpopulation. (F) RNA velocity analysis is used to assess the dynamic changes in cell states. (G) Pseudotime analysis of neuroblast differentiation trajectories between 7dpi and sham. (H) The expression profile heatmap displays the differentially expressed molecules between 7dpi and sham.

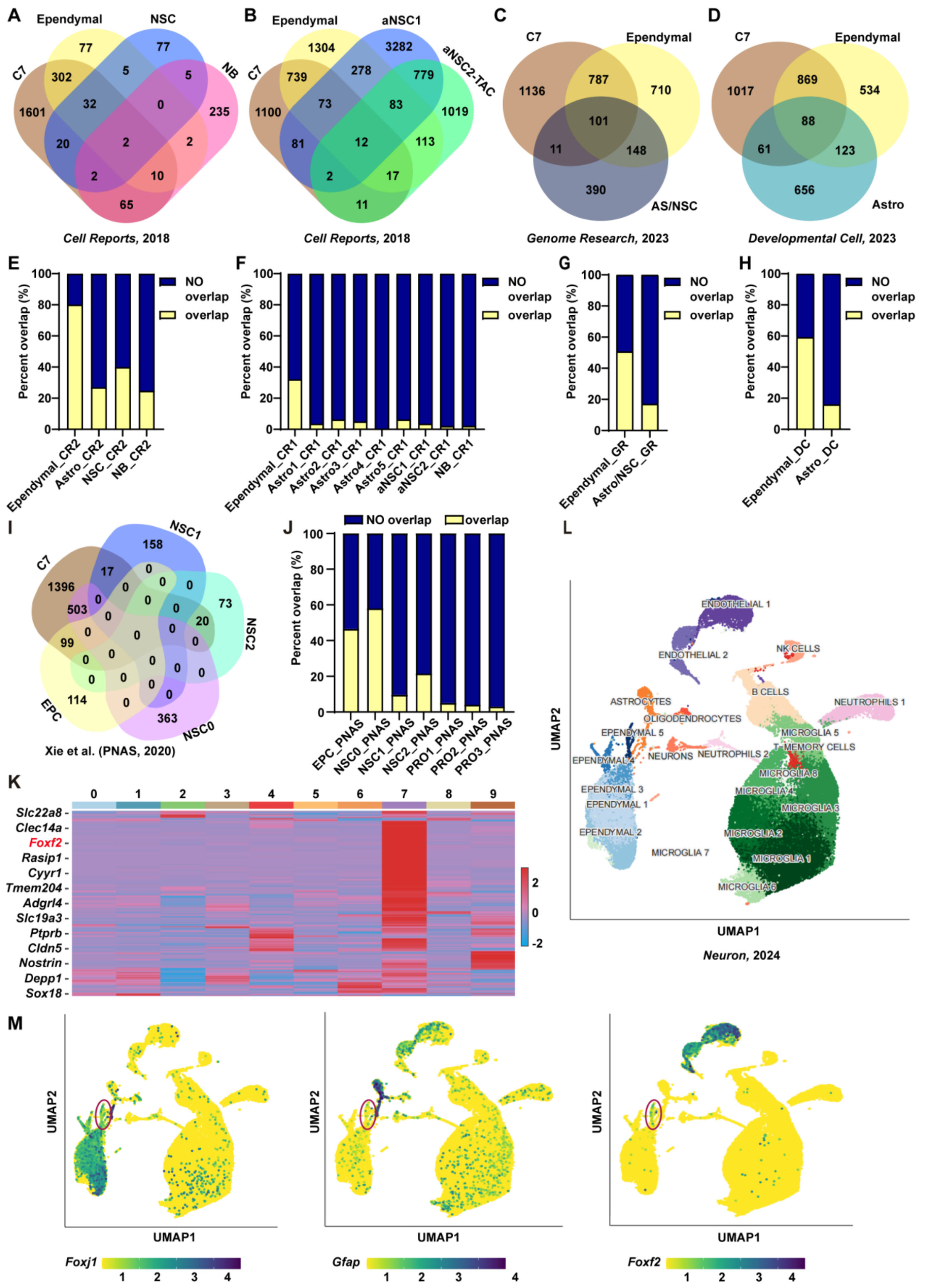

Fig. S3. Comparative gene expression profiling between cluster 7 and ependymal cells reported in other literature

(A) Comparative gene expression profiling between cluster 7 (C7) and the ependymal cell, neural stem cell (NSC) or neuroblasts (NB) subpopulations dataset published by Zywitza et al. (*Cell Reports*, 2018) shows that C7 shares the most similar expression profiles with the

ependymal cell. (B-D)Comparative gene expression profiling between C7 and the ependymal cell or NSC subpopulations dataset published by Mizrak et al. (*Cell Reports*, 2018, E); Yang et al.(*Genome Research*, 2023, F); and Albors et al. (*Developmental Cell*, 2023).(E-H) Comparative gene expression profiling between C7 and the ependymal cell or nerual stem cell (NSC) subpopulations dataset published by Zywitza et al. (*Cell Reports*, 2018, CR1); Mizrak et al. (*Cell Reports*, 2018, CR2); Yang et al.(*Genome Research*, 2023, GR); and Albors et al. (*Developmental Cell*, 2023, DC).(I-J) Comparative gene expression profiling between cluster 47 and the ependymal cell subpopulations dataset published by Xie et al. (*PNAS*, 2020) shows that C7 shares the most similar expression profiles with the neural stem cell subpopulation NSC0 and the endothelial progenitor cell (EPC) subpopulation. Additionally, the characteristic molecule *Foxf2* is also identified in the EPC subpopulation in that report. (K) The expressed genes in EPC, when compared with all subpopulations in Fig. 1, show the most similar expression profile with the highly expressed genes of C7. (L-M) Comparative analysis of GFAP^{+} FOXF2^{+} double positive cell expression characteristics with the latest TBI injury model sequencing data.

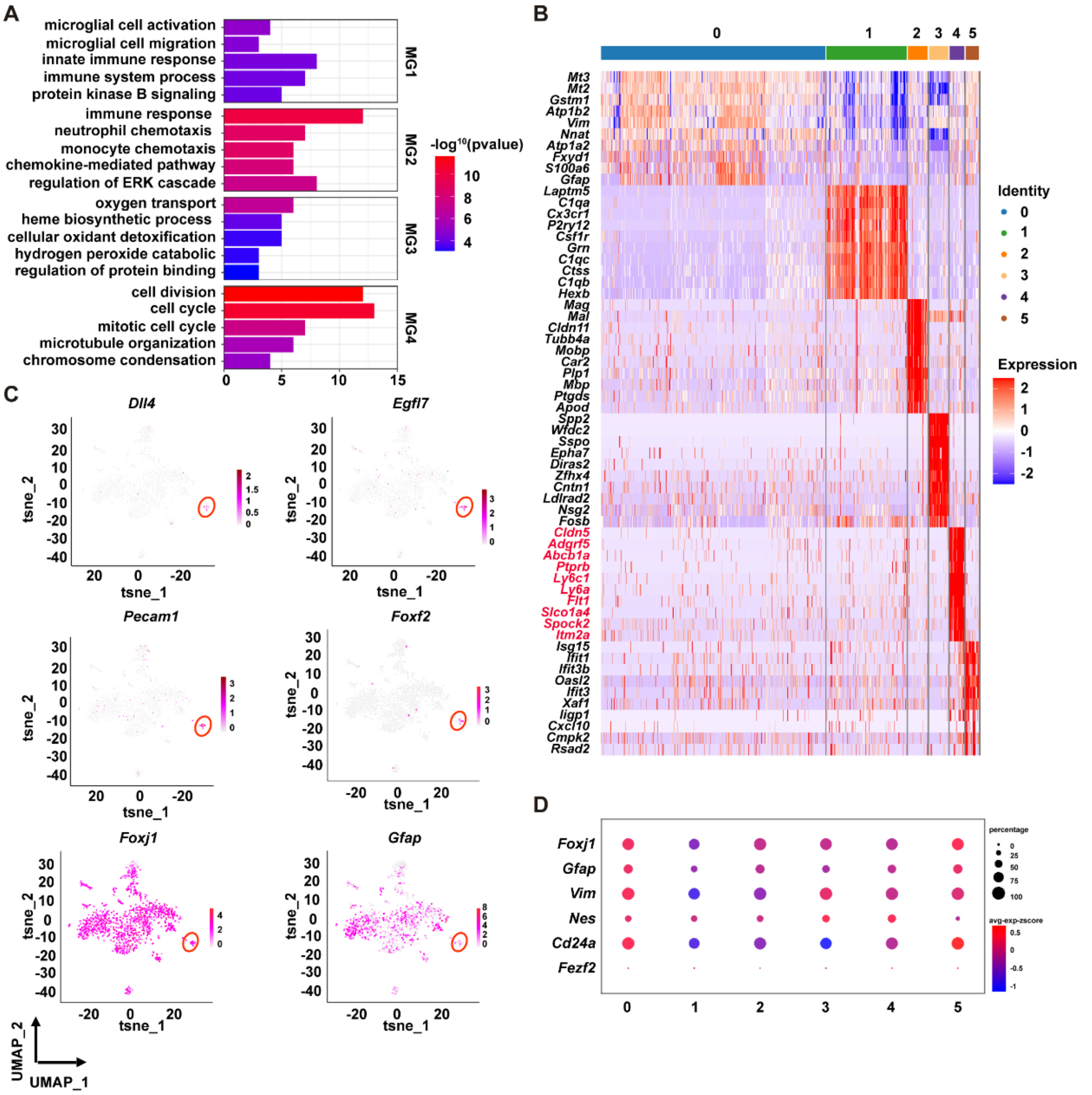

Fig. S4. Functional and molecular characterization of cellular subpopulations in the infarcted region

(A) Biological process analyses for each microglial cluster. MG1-4, microglial cluster 1-4. (B) Heat map illustrating the characteristic expression molecules of individual cell subsets. (C) Scatter plots showing molecules associated with angiogenesis in cluster 4. (D) The results show that cluster 47 in Fig. 1 shares the most similar expression profile with cluster 4 in Fig. 2, with 778 overlapping genes. (E) The top 50 highly expressed genes in cluster 4, when compared with all subpopulations in Fig. 1, show the most similar expression profile with the highly expressed genes of cluster 47. (F) Comparison of expression profiles of C11 ependymal subgroups with previously reported E1, E2, and E3 markers

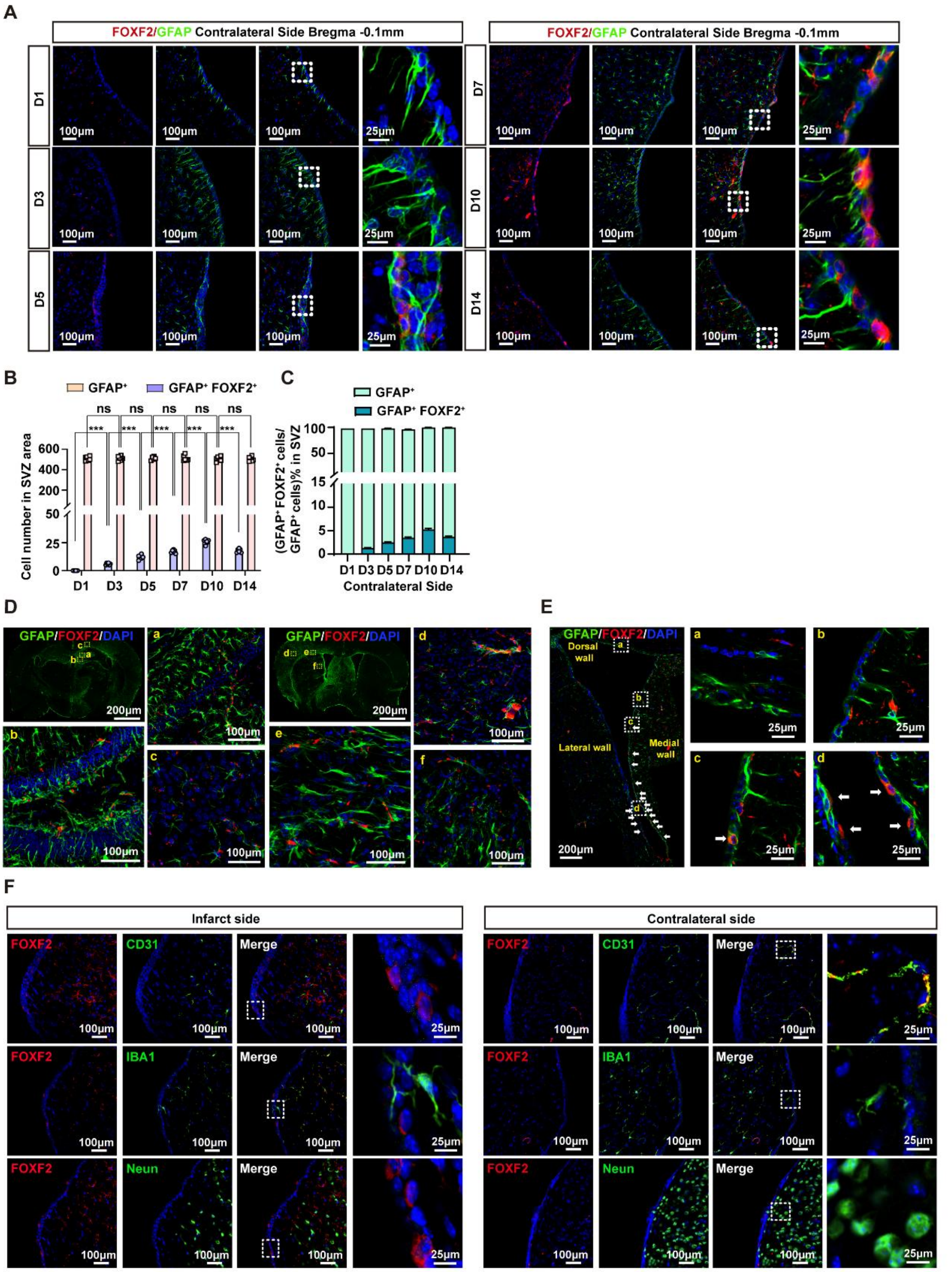

Fig. S5. Specificity validation of GFAP and FOXF2 antibodies

(A) Representative immunofluorescence images of FOXF2 and GFAP expression on the contralateral side of the V-SVZ region at different time points after I/R. n = 6, magnification 400×; the yellow triangle indicates GFAP⁺ FOXF2⁺cells; the dotted boxes indicate the colabeled areas of the cells that are magnified. (B) Quantitative counting of GFAP⁺ FOXF2⁺ cells in the V-SVZ region at different time points after I/R; t-test, ***p* < 0.05, ****p* < 0.01, *****p* < 0.001; ns, not significant. (C) Quantitative comparison of the proportion of GFAP+ FOXF2+ cells in the V-SVZ region at different time points after I/R. (D) Distribution characteristics of GFAP⁺ FOXF2⁺ cells

in the whole brain after I/R except for the SVZ: a, hippocampal fissure region; b, dentate gyrus region; c, cingulate cortex region; d, primary somatosensory cortex region; e, external capsule region; f, caudate putamen region. (E) Distribution characteristics of $GFAP^{+}$ $FOXF2^{+}$ cells in the V-SVZ after I/R. (F) Representative immunofluorescence image of FOXF2 colocalized with other neuronal markers in the SVZ region.

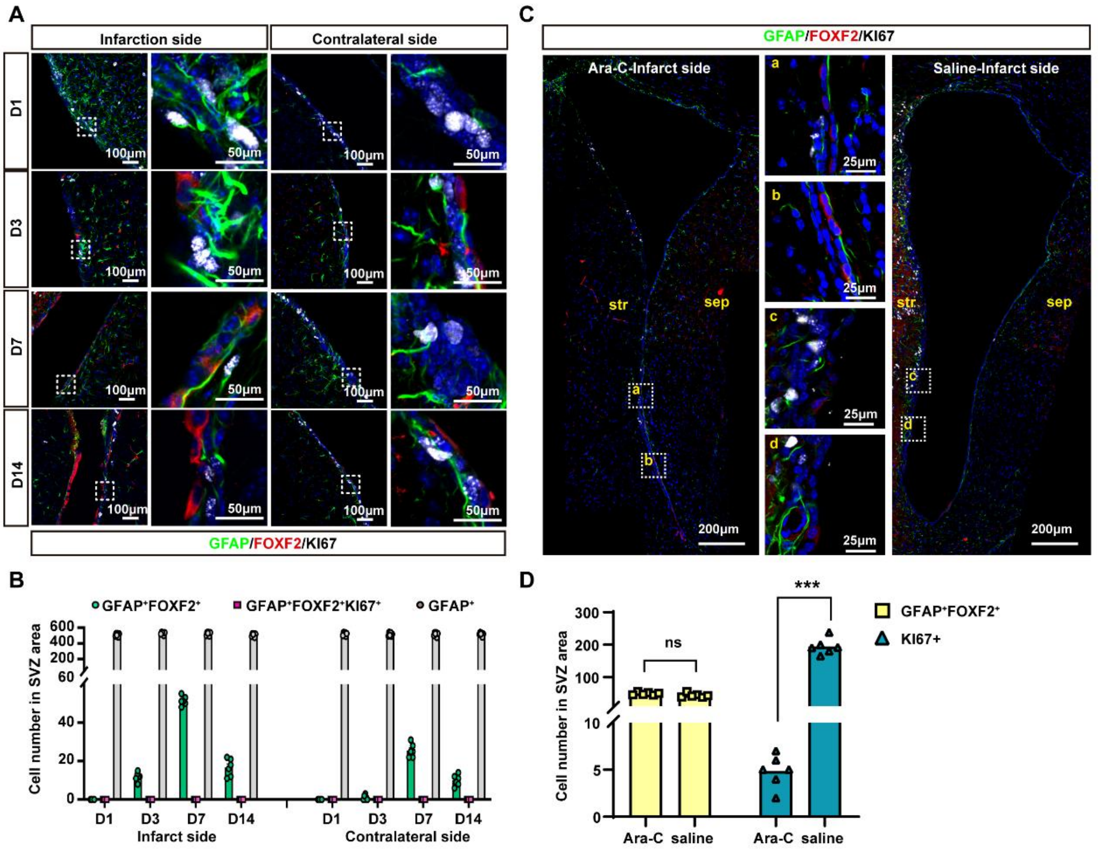

Fig. S6. Experimental evidence demonstrated that dual GFAP$^+$FOXF2$^+$ cells lack proliferative capacity

(A)Cell proliferation marker KI67 is not co-labeled with FOXF2$^+$GFAP$^+$ cells in the V-SVZ region after I/R. The dotted boxes indicate the co-labeled areas of the cells that are magnified, n = 6 mice per group. (B)Count the number of cells expressing GFAP$^+$FOXF2$^+$KI67$^+$, GFAP$^+$FOXF2$^+$ and GFAP$^+$ in the infarct and contralateral sides. (C) The distribution of cell proliferation markers KI67$^+$ and FOXF2$^+$GFP$^+$ cells in the SVZ region after treatment with Ara-C, with n=6 mice in each group. (D) Count the number of GFAP$^+$FOXF2$^+$ and KI67$^+$ cells in the SVZ region, *t*-test, ****$p < 0.001$, ns, no significance.

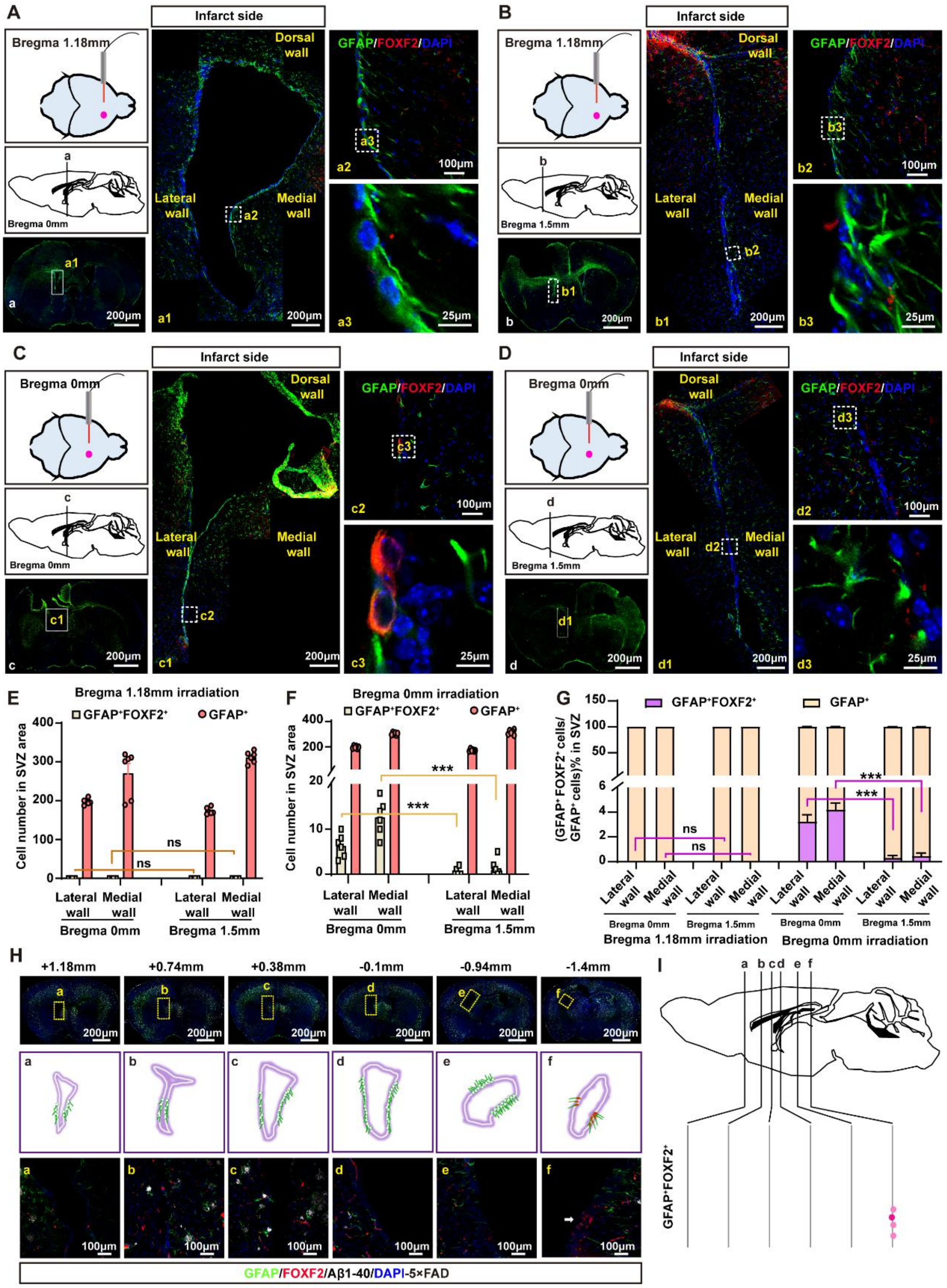

Fig. S7. GFAP⁺FOXF2⁺ cells are expressed in the V-SVZ region in other disease models

(A-D) Photothrombotic models at Bregma of -0.1mm and 1.18 mm to evaluate the double-positive cells on near and far from the injury locations. (E-F) Count the number of FOXF2⁺GFAP⁺ cells in two regions : medial wall and lateral wall. (G) Calculate the proportion of FOXF2⁺ cells among all GFAP⁺ cells in the different V-SVZ region.(H) Detection of FOXF2⁺GFAP⁺ cells in the SVZ region of the Alzheimer's disease (AD) model(I) Count the number of GFAP⁺FOXF2⁺ cells in the SVZ region of the AD model.

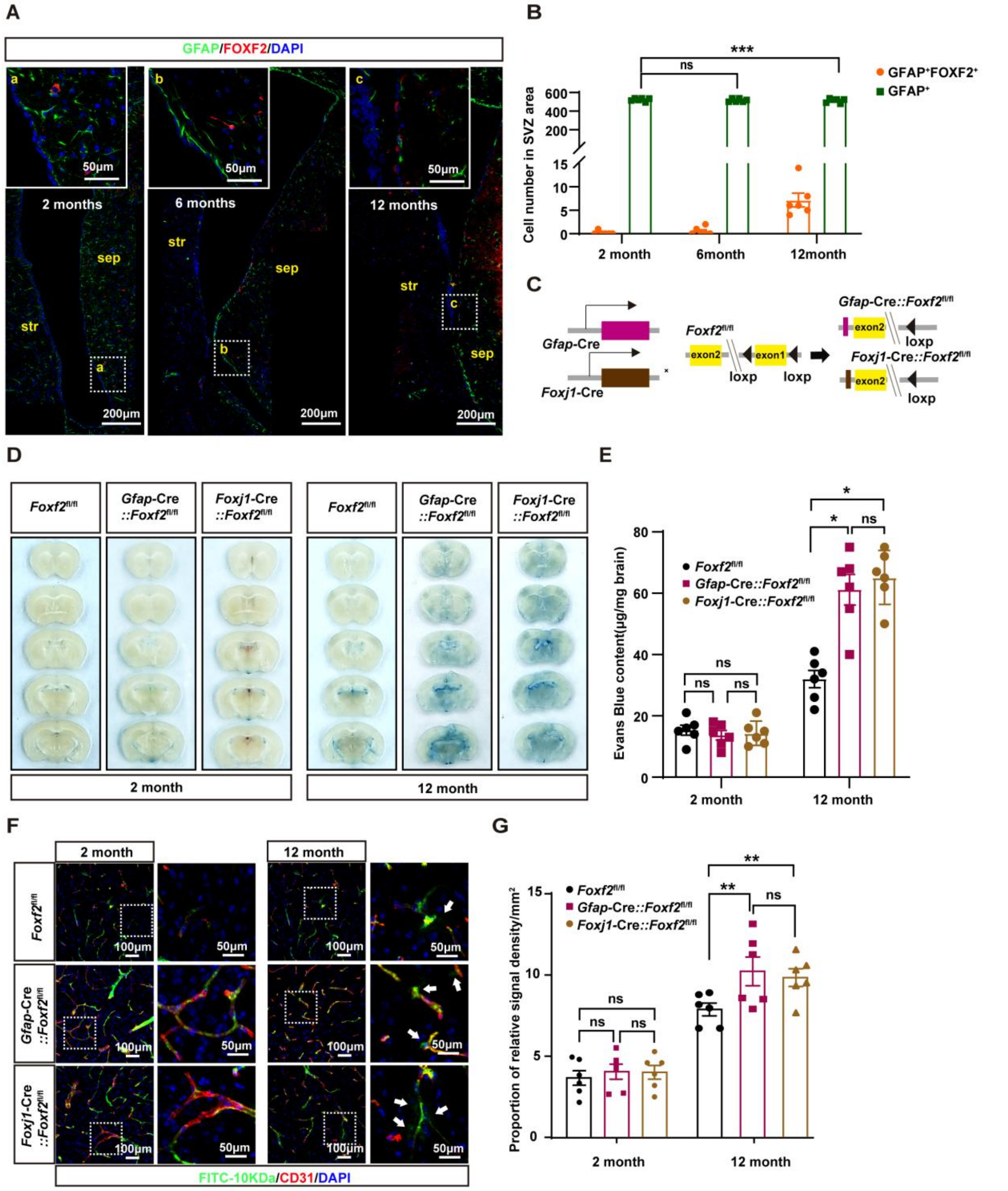

Fig. S8. Comparative effects of *Gfap*-Cre and *Foxj1*-Cre driven *Foxf2* deletion on vascular leakage under physiological and aging conditions

(A-B) Quantitative comparison of the number of GFAP^{+}FOXF2^{+} cells in the V-SVZ region between 2-month-old, 6-month-old and 12-month-old mice under physiological conditions, *t*-test, ****p* < 0.001, ns, no significance, n=6. (C) Construction plan for *Foxf2* CKO and *Foxj1* CKO mice: *Foxf2*$^{fl/fl}$ mice were hybridized with *Gfap*-Cre and *Foxj1*-Cre mice, respectively, to obtain *Gfap*-Cre::*Foxf2*$^{fl/fl}$ and *Foxj1*-Cre::*foxf2*$^{fl/fl}$ conditional deletion mice. (D) Evans blue staining results for 2-month-old and 12-month-old *Foxf2*-CKO mice and control (*Foxf2*$^{fl/fl}$), n = 6 each. (E) Quantitative analysis of the data in (D), *t*-test, **p* < 0.05, ns, no significance. (F) Representative images of 10kDa FITC (green) co-immunostained with anti-CD31 in *Foxf2*-CKO mice and control (*Foxf2*$^{fl/fl}$). (G) Quantitative analysis of the data in (F), *t*-test, ***p* < 0.01, ns, no significance.

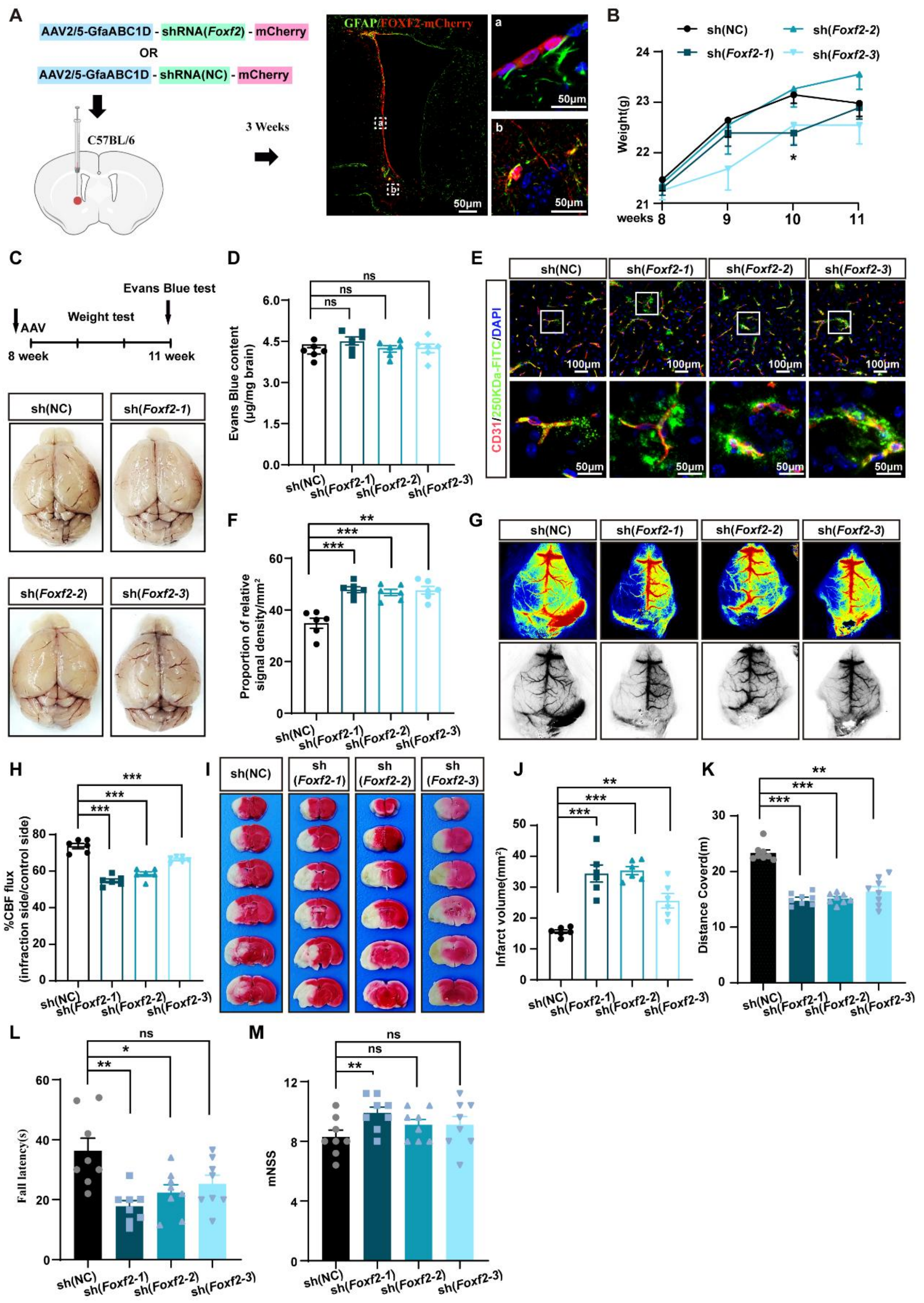

Fig. S9. AAV-mediated knockdown of FOXF2 in GFAP^{+} cells exacerbates BBB injury after I/R

(A) Description of AAV virus construction and injection protocols. Abbreviated as AAV2/5-gfaABC1D-shRNA(*Foxf2*)-mCherry and AAV2/5-gfaABC1D-shRNA(NC)-mCherry respectively as sh(*Foxf2*), and sh(NC). (B) Statistical analysis of body weight measurements in mice post AAV virus injection, n = 8. (C) Evans blue assay results for sh(NC) and sh(*Foxf2*) groups (n =

6 each). (D) Quantitative analysis of the data in (C). (E) Representative images of 250kDa FITC (green) co-immunostained with anti-CD31 in sh(NC) and sh(*Foxf2*) mice following I/R. (F) Quantitative analysis of the data in (E), *t*-test, $**p < 0.01$, $***p < 0.001$, n=6. (G) Representative images and quantification of laser Doppler measurement of blood flow signals in sh(NC) and sh(*Foxf2*) groups following I/R. (H) Quantitative analysis of the data in (G), *t*-test, $**p < 0.01$, $***p < 0.001$, n=6. (I) Representative photographs of coronal brain sections of sh(NC) and sh(*Foxf2*) groups stained with TTC following MCAO and relative infarct volume. (J) Quantitative analysis of the data in (G), *t*-test, $**p < 0.01$,$***p < 0.001$, n=6. (K) Open field test (OFT) , Rotarod test and mNSS test of sh(NC) and sh(*Foxf2*) mice after MCAO, *t*-test, $**p < 0.05$, $**p < 0.01$, $***p < 0.001$, n=8.

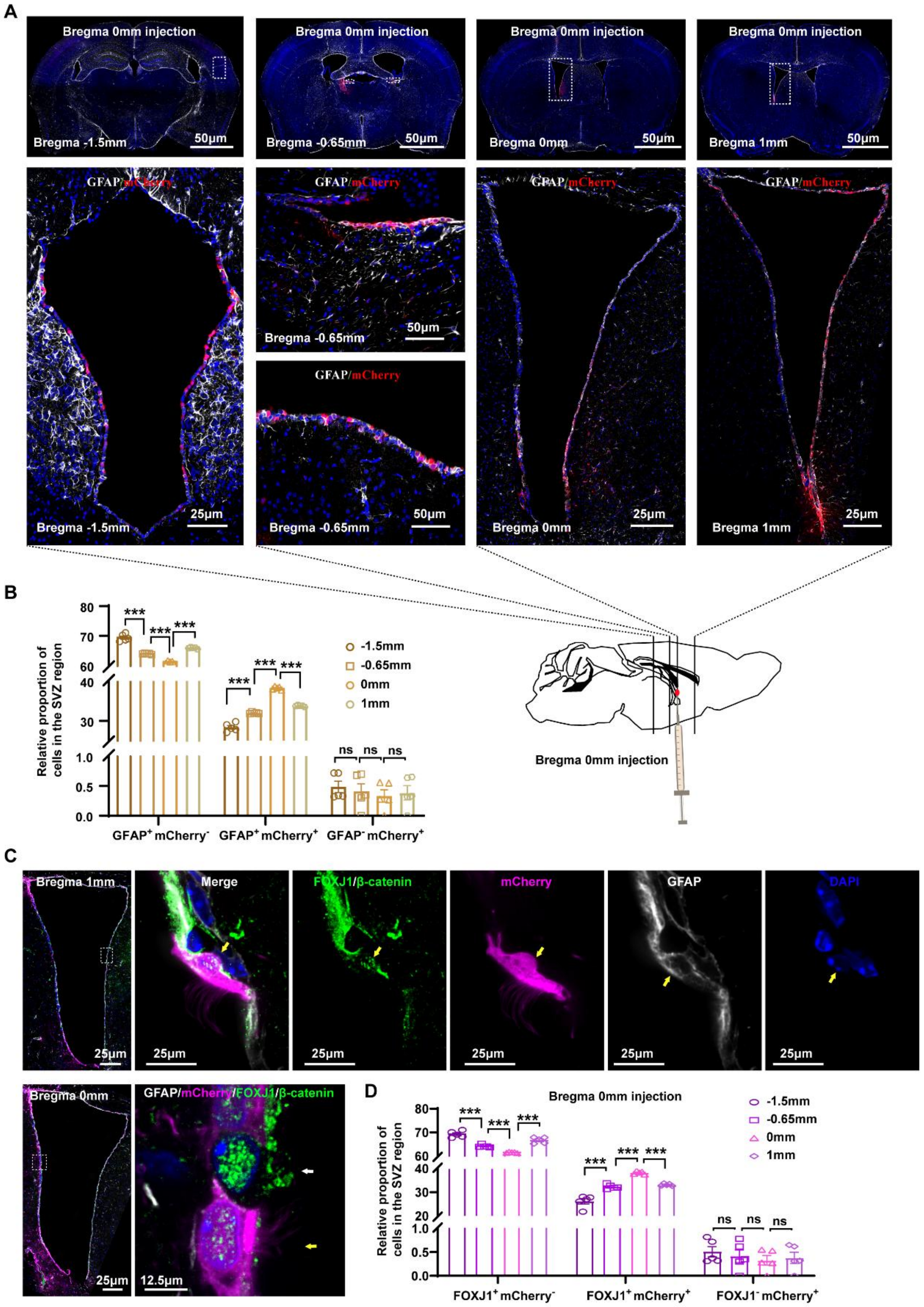

Fig. S10 Evaluation of AAV infection efficiency

(A-B) Quantitative comparison of the proportion of GFAP$^+$ mCherry$^+$ cells, GFAP$^+$ mCherry$^-$ cells and GFAP$^-$ mCherry$^+$ cells in the SVZ region at different levels of mouse brain. (C-D) Quantitative comparison of the proportion of FOXJ1$^+$ mCherry$^+$ (yellow arrow) cells, FOXJ1$^+$ mCherry$^-$ (white arrow) cells and FOXJ1$^-$ mCherry$^+$ cells in the SVZ region at different levels of mouse brain, *t*-test, ***$p < 0.001$, ns, no significance, n=5.

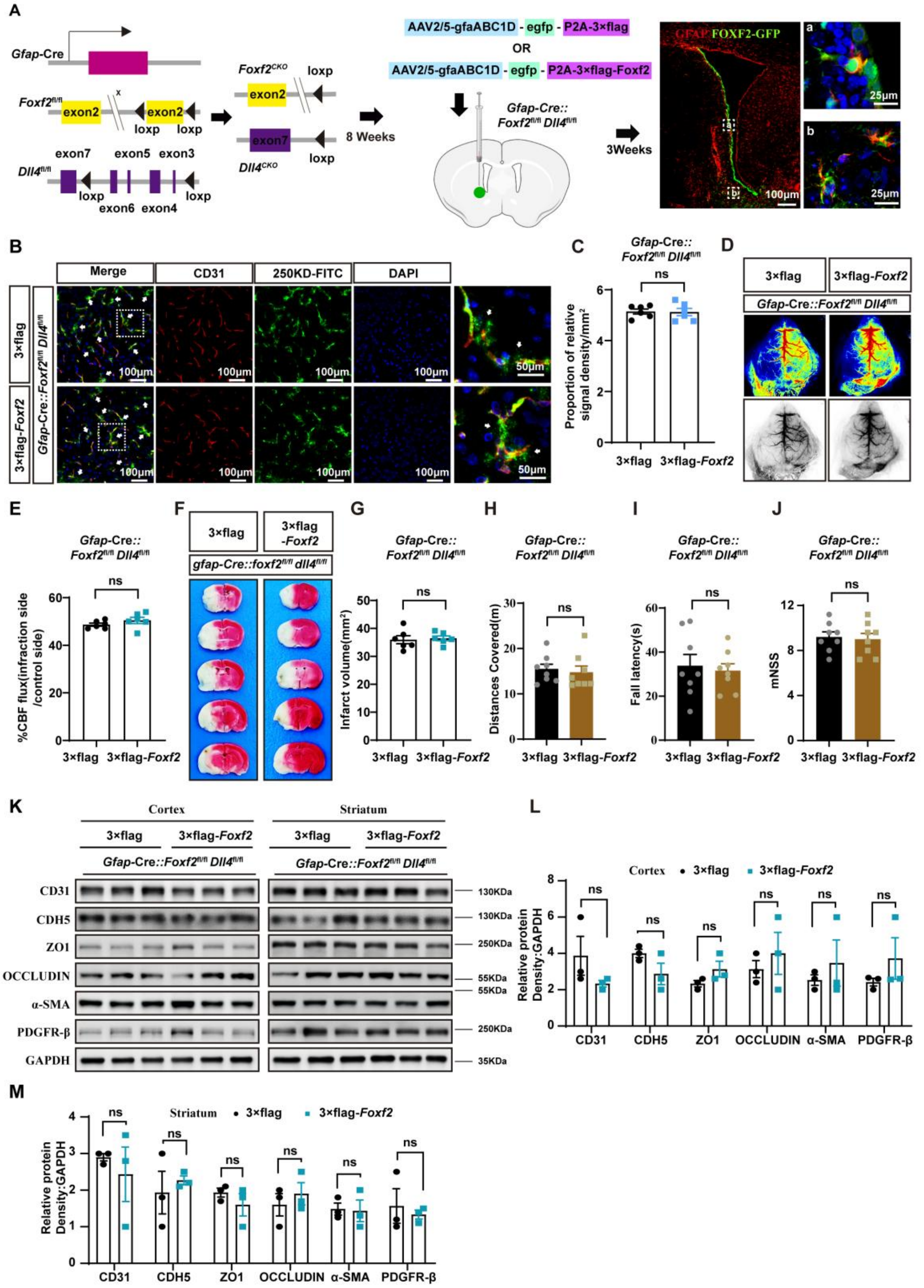

Fig. S11. GFAP^{+}FOXF2^{+} ependymal cells is dependent on the *Dll4-Notch* pathway to regulate vascular permeability.

(A) Experimental flowchart for the construction of *Gfap*-Cre::*Foxf2*$^{fl/fl}$*Dll4*$^{fl/fl}$ (dobule-CKO) mice and the injection of *Foxf2* overexpression AAV. (B) Representative image of co immunostaining of 250kDa FITC (green) and anti-CD31 in dobule-CKO *and Foxf2* overexpressing mice after I/R. (C) Quantitative analysis was performed on the data in (B), t-test, ns, no significance. (D)

Representative images and quantification of laser Doppler measurement of blood flow signals in dobule-CKO *and Foxf2* overexpressing mice following I/R, n=6 each. (E) Quantitative analysis of the data in (D), *t*-test, ns, no significance. (F) Representative photographs of coronal brain sections of dobule-CKO *and Foxf2* overexpressing mice stained with TTC following I/R and relative infarct volume, n=6 mice per group. (G) Quantitative analysis of the data in (F), *t*-test, ns, no significance. (H-J) Open field test (OFT) , Rotarod test and mNSS test of dobule-CKO *and Foxf2* overexpressing mice after MCAO, *t*-test, ns, no significance, n=8. (K) Comparison of expression levels of vascular permeability-related proteins between the dobule-CKO *and Foxf2* overexpressing mice, n=3 each. (L-M) Quantitative analysis of the data in (H), *t*-test, ns, no significance.

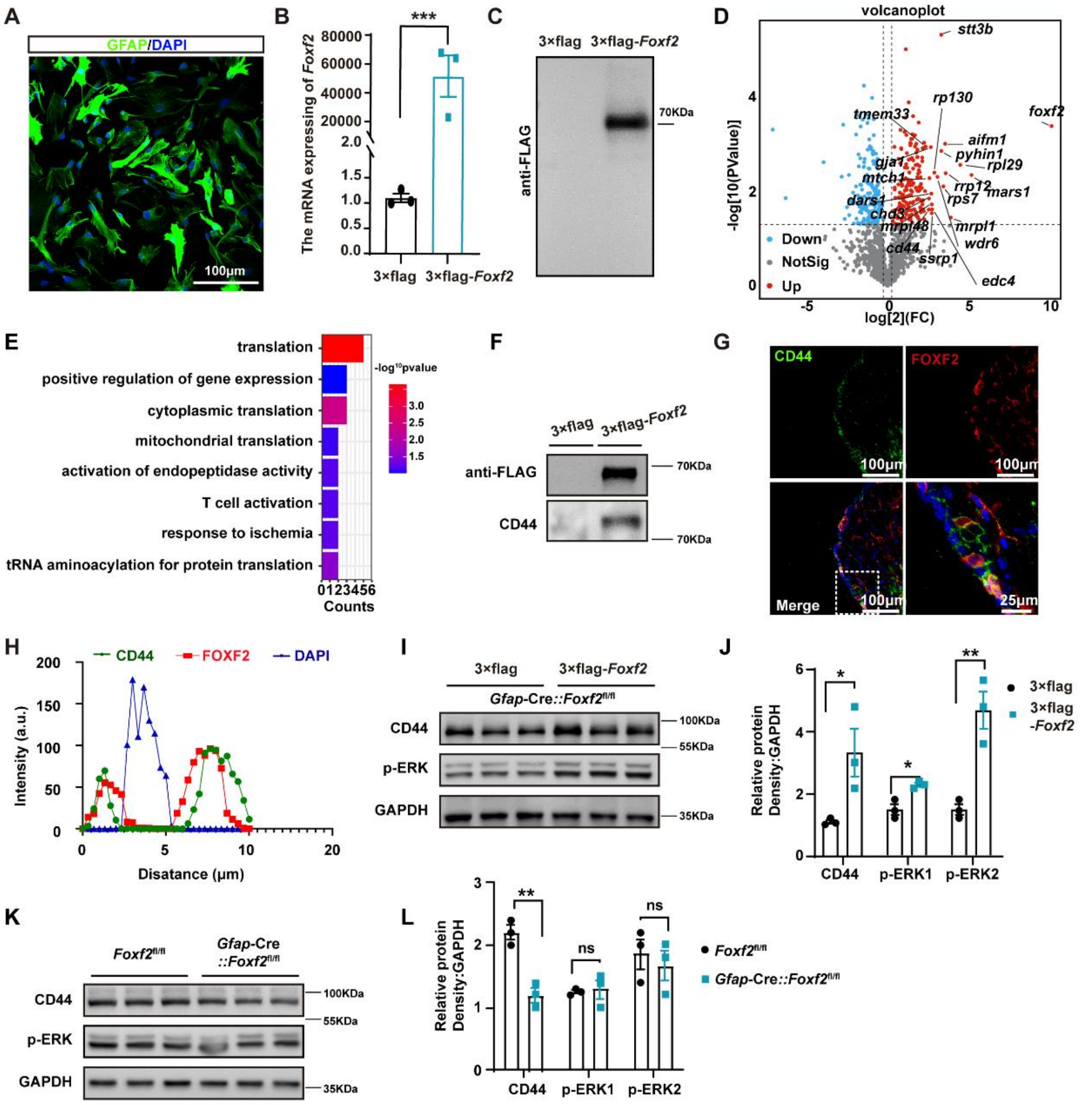

Fig. S12. FOXF2 promotes DLL4 expression by promoting ERK phosphorylation upon binding to CD44

(A) Isolation and purification of primary astrocytes from newborn mouse brain tissue. (B-C) Evaluation of *Foxf2* expression after transfection of primary astrocytes, t-test, * * * p<0.001.

(D) The volcano plot displays proteins significantly enriched in interaction with *Foxf2* virus. (E) Functional clustering analysis of these 20 proteins identified in (D) reveals three proteins enriched in gene regulatory pathways. (F) Immunoprecipitation demonstrates an interaction between FOXF2 and CD44. (G) Immunofluorescence representative images show the expression of FOXF2 and CD44 in the SVZ region following stroke. (H) The spatial location of protein expression in cells. (I-L) The Western blot and its quantitative results show a significant increase in the expression levels of CD44, the downstream molecule ERK phosphorylation and DLL4 in the CD44 pathway, in mice overexpressing *Foxf2, t*-test, *$p < 0.05$, **$p < 0.01$.
